# A Structural Investigation of the Interaction between a GC-376-Based Peptidomimetic PROTAC and Its Precursor with the Viral Main Protease of Coxsackievirus B3

**DOI:** 10.3390/biom14101260

**Published:** 2024-10-06

**Authors:** Alessia De Santis, Deborah Grifagni, Andrea Orsetti, Elena Lenci, Antonio Rosato, Mariapina D’Onofrio, Andrea Trabocchi, Simone Ciofi-Baffoni, Francesca Cantini, Vito Calderone

**Affiliations:** 1 Magnetic Resonance Center CERM, University of Florence, Via Luigi Sacconi 6, Sesto Fiorentino, 50019 Florence, Italy; desantis@cerm.unifi.it (A.D.S.); grifagni@cerm.unifi.it (D.G.); a.orsetti@uu.nl (A.O.); rosato@cerm.unifi.it (A.R.); ciofi@cerm.unifi.it (S.C.-B.); 2 Department of Chemistry, University of Florence, Via della Lastruccia 3-13, Sesto Fiorentino, 50019 Florence, Italy; elena.lenci@unifi.it (E.L.); andrea.trabocchi@unifi.it (A.T.); 3 Department of Biotechnology, University of Verona, Strada le Grazie 15, 37134 Verona, Italy; mariapina.donofrio@univr.it

**Keywords:** PROTAC, viral main protease, 3-chymotrypsin-like protease, Coxsackievirus B3, SARS-CoV-2, GC-376

## Abstract

The conservation of the main protease in viral genomes, combined with the absence of a homologous protease in humans, makes this enzyme family an ideal target for developing broad-spectrum antiviral drugs with minimized host toxicity. GC-376, a peptidomimetic 3CL protease inhibitor, has shown significant efficacy against coronaviruses. Recently, a GC-376-based PROTAC was developed to target and induce the proteasome-mediated degradation of the dimeric SARS-CoV-2 3CL^Pro^ protein. Extending this approach, the current study investigates the application of the GC-376 PROTAC to the 3C^Pro^ protease of enteroviruses, specifically characterizing its interaction with CVB3 3C^Pro^ through X-ray crystallography, NMR (Nuclear Magnetic Resonance) and biochemical techniques. The crystal structure of CVB3 3C^Pro^ bound to the GC-376 PROTAC precursor was obtained at 1.9 Å resolution. The crystallographic data show that there are some changes between the binding of CVB3 3C^Pro^ and SARS-CoV-2 3CL^Pro^, but the overall similarity is strong (RMSD on C-alpha 0.3 Å). The most notable variation is the orientation of the benzyloxycarbonyl group of GC-376 with the S4 subsite of the proteases. NMR backbone assignment of CVB3 3C^Pro^ bound and unbound to the GC-376 PROTAC precursor (80% and 97%, respectively) was obtained. This information complemented the investigation, by NMR, of the interaction of CVB3 3C^Pro^ with the GC-376 PROTAC, and its precursor allows us to define that the GC-376 PROTAC binds to CVB3 3C^Pro^ in a mode very similar to that of the precursor. The NMR relaxation data indicate that a quench of dynamics of a large part of the protein backbone involving the substrate-binding site and surrounding regions occurs upon GC-376 PROTAC precursor binding. This suggests that the substrate cavity, by sampling different backbone conformations in the absence of the substrate, is able to select the suitable one necessary to covalently bind the substrate, this being the latter reaction, which is the fundamental step required to functionally activate the enzymatic reaction. The inhibition activity assay showed inhibition potency in the micromolar range for GC-376 PROTAC and its precursor. Overall, we can conclude that the GC-376 PROTAC fits well within the binding sites of both proteases, demonstrating its potential as a broad-spectrum antiviral agent.

## 1. Introduction

Cells use the ubiquitin–proteasome system to degrade unwanted or misfolded cellular proteins [1,2]. To perform this, the system targets proteins for proteasome-mediated destruction by polyubiquitinating them. This process involves the sequential action of the E1 ubiquitin-activating enzyme, the E2 ubiquitin-conjugating enzyme and the E3 ubiquitin–protein ligase [3,4,5,6]. The E3 ligase is responsible for attaching multiple ubiquitin molecules to lysine residues at recognition sites in the protein to be degraded. This post-translational modification serves as a signal for proteolytic action by the 26S proteasome [7]. This cellular degradation system has been exploited by PROteolysis TArgeting Chimera (PROTAC) technology to modulate the cell levels of target proteins. PROTAC has proven highly effective in targeting proteins associated with cancer, leading to the initiation of clinical investigations for over a dozen drug candidates [8,9,10]. The utilization of the PROTAC technology as antiviral agent, although more limited, has recently grown. In the last year, it has indeed received a strong impetus with four works, including one from our group, that delineated the use of different PROTAC molecules against the 3-chymotrypsin-like protease of SARS-CoV-2 (SARS-CoV-2 3CL^Pro^, hereafter) [11,12,13,14]. In our previous study [12], we have synthesized a PROTAC bifunctional molecule. This molecule conjugates a unit derived from the GC-376 inhibitor, which is able to recognize the active site of SARS-CoV-2 3CL^Pro^ [15,16], with the pomalidomide ligand, which is able to select Cereblon (CRBN) E3 ligase [17,18,19,20,21]. The GC-376 inhibitor and the pomalidomide ligand are joined through a piperazine–piperidine linker. We showed that this PROTAC molecule is able to interact with SARS-CoV-2 3CL^Pro^, forming a reversible covalent bond between the Cβ carbon of the α,β-unsaturated amide moiety and the catalytic Cys145 sulfur atom of the protein. Moreover, we observed that our PROTAC molecule reduces protein levels of SARS-CoV-2 3CL^Pro^ in cultured cells without affecting cell viability. This demonstrates that a peptidomimetic-based PROTAC approach can attack viral infections within those coronaviruses displaying a high degree of structural similarity in the active site of their 3CL^Pro^ with that of SARS-CoV-2 3CL^Pro^ [22].

Proteases are also important for the replication of viruses other than coronaviruses [23]. Among them, we have the proteases encoded by the 3C region of the enteroviruses genome (3C^Pro^, hereafter), such as Coxsackievirus B3 (CVB3), EV-D68, EV-A71 and Coxsackievirus A16 [24,25,26]. All these enteroviral pathogens are part of the picornavirus family [27]. Infection with some of these viruses can lead to serious outcomes and cause clinical disease much more frequently than coronaviruses. As an example, infection with Coxsackievirus B3, a nonenveloped single-stranded (+) RNA enterovirus, is the most common reason for viral myocarditis and sudden cardiac death, within the enterovirus genus, with a very malicious disease pattern [28,29]. No effective therapeutic strategy for the prevention and treatment of these diseases is nowadays available, and enterovirus disease has a medical impact, with newborn infants and young children being at risk for septic-like disease [30]. The fact that all these virally encoded proteases from coronaviruses and enteroviruses perform essential functions in the viral replication cycle by cleaving both viral and host targets [31,32], as well as the fact that there is no known human protease with a specificity for Gln at the cleavage site of the substrate, has pushed the scientific community towards the development of a commercially viable antiviral drug targeting both of these virus families [33]. This approach is made viable because coronaviral 3CL^Pro^ exhibits a high degree of structural similarity across 3C^Pro^ of the picornavirus family [23]. Crystal structures of 3CL^Pro^ from both alpha and beta coronaviruses [34,35] revealed that two of the three domains of these enzymes together resemble the chymotrypsin-like fold of enteroviral 3C^Pro^, with the exception of an additional α-helical domain that is involved in the dimerization of coronaviral proteases. While this dimerization is essential for the catalytic activity of 3CL^Pro^, the enteroviral 3C^pro^ functions as a monomer [34]. In addition, the enteroviral 3C^Pro^ maintains a classical Cys···His···Glu/Asp catalytic triad, whereas the coronaviral 3CL^Pro^ only has a Cys···His dyad [34]. Nevertheless, these two types of proteases share common features, in particular their almost-absolute requirement for Gln in the P1 position of the substrate and space for only small amino-acid residues such as Gly, Ala, or Ser in the P1′ position of the substrate, indicating that the coronaviral 3CL^Pro^ and the enteroviral 3C^Pro^ can be a common target for the design of broad-spectrum antiviral compounds [34].

**Figure 1:**
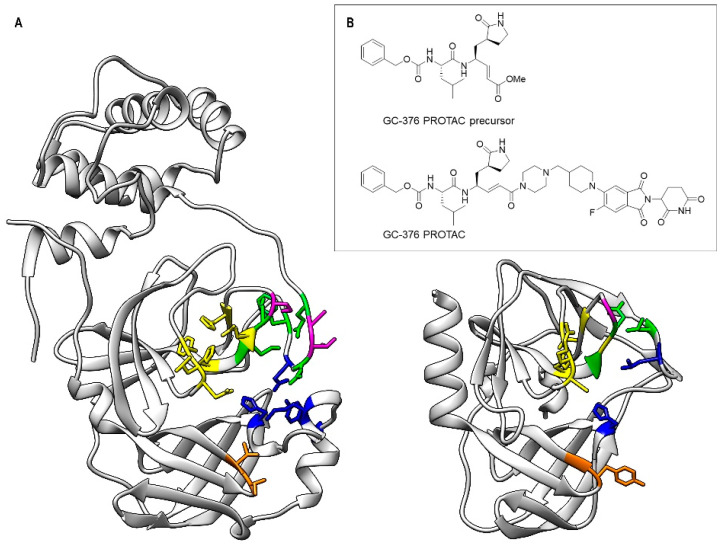
Comparison of the substrate-binding subsites (S1, S1′, S2, S4 and S5) of SARS-CoV-2 3CL^Pro^ (left) and of CVB3 3C^Pro^ (right) (**A**) and 2D structure of the GC-376 PROTAC and its precursor (**B**). In the two protein structures, the backbone and side chains of the residues of the subsites are color-coded (S1—yellow, S1′—orange, S2—blue, S4—green, S5—purple).

3CL^Pro^ has several subsites (“S”) for substrate binding, which are identified by the Schechter and Berger (1967) nomenclature [36]. These include S1 (Phe140, Leu141, Asn142, His163, Glu166 and His172), S1′ (Thr24 and Thr25), S2 (His41, Met49, Tyr54, Met165 and Asp187), S4 (Leu167, Phe185, Gln189 and Gln192) and S5 (Pro168, Thr190 and Ala191), and they are partially maintained in 3C^Pro^ [37] (Figure 1A). The latter has a very similar backbone structural arrangement in the S1 and S1′ subsites although it has different residues (Thr142, Arg143, Ala144, Gln146, His161, Gly164 and Gly169 in S1 and Tyr22 and Gly23 in S1′), but it has an open and shallow S2 site with only His40 as conserved residue [38,39] (Figure 1A). The S4 and S5 subsites in 3C^Pro^ are also structurally different and more open compared to 3CL^Pro^. This difference arises because residues Gln189, Thr190, Ala191 and Gln192, which are located on the loop connecting domains I and II to domain III in 3CL^Pro^, are absent in 3C^Pro^. Notably, 3C^Pro^ lacks domain III, which is responsible for enzyme dimerization in 3CL^Pro^ (Figure 1A). Despite the structural differences present among the various subsites, broad-spectrum inhibitors of both 3CL^Pro^ and 3C^Pro^ have been largely documented in the literature [40,41,42,43,44,45,46,47,48,49], encouraging us to explore the capability of the PROTAC molecule previously designed by us against SARS-CoV-2 3CL^Pro^ to also target enteroviral 3C^Pro^. To this aim, we have employed X-ray crystallography and solution NMR to characterize the interaction of 3C^Pro^ from Coxsackievirus B3 (CVB3 3C^Pro^, hereafter) with the peptidomimetic PROTAC molecule previously selected on SARS-CoV-2 3CL^Pro^ (GC-376 PROTAC) as well as with its precursor (GC-376 PROTAC precursor), i.e., a dipeptidyl protease ligand (Figure 1B) [12]. We have also compared the inhibitory activity of the GC-376 PROTAC precursor against CVB3 3C^Pro^ with that obtained in the case of the SARS-CoV-2 3CL^Pro^ enzyme.

## 2. Materials and Methods

### 2.1. Expression and Purification of CVB3 3C^Pro^

CVB3 3C^Pro^ was expressed and purified using a modification of Fili et al.’s protocol [50]. In detail, the pET-24a(+) plasmid encoding the CVB3 3C^Pro^ protein with an hexahistidine-coding sequence at the C-terminus was used to transform competent BL21(DE3)pLysS *E. coli* cells. A single colony from the transformation plate was picked to inoculate a 50 mL preculture in Luria–Bertani (LB) medium containing 50 μg mL^−1^ kanamycin and 35 μg mL^−1^ chloramphenicol. After overnight incubation at 37 °C, 20 mL of the preculture was used to inoculate 1 L LB medium containing 50 μg mL^−1^ kanamycin and 35 μg mL^−1^ chloramphenicol. When an optical absorption of 0.6 (at a wavelength of 600 nm) was reached, isopropyl β-D-1-thiogalactopyranoside (IPTG) was added to a final concentration of 1 mM to induce overexpression. The culture was then left overnight at 23 °C and then centrifuged at 5500 rpm for 20 min at 4 °C with a JA-10 Beckman rotor. The resulting pellet was resuspended in a 20 mL lysis buffer consisting of 50 mM Tris-HCl, 300 mM NaCl, 10 mM imidazole, 5% glycerol and 0.1% Triton-X pH 7.7. The cell suspension was lysed by one cycle of freezing (−80 °C) and thawing (+10–15 °C) and sonication upon the addition of 20 mL lysis buffer, 2 mM DTT and lysozyme (0.15 mg mL^−1^). The soluble cellular fraction was obtained by centrifugation at 4 °C at 40,000 rpm for 35 min with a 70Ti Beckman rotor. Then, the supernatant containing soluble CVB3 3C^Pro^ was loaded for Immobilized Metal Affinity Chromatography (IMAC) purification by using a HisTrap FF 5mL column, which was previously equilibrated with 50 mM Tris-HCl, 300 mM NaCl, 30 mM imidazole and 1 mM DTT pH 7.7 as a binding buffer. After lysate loading, the column was washed with 20 column volumes of the same buffer, and elution was executed with 10 column volumes of 50 mM Tris-HCl, 300 mM NaCl, 500 mM imidazole and 1 mM DTT pH 7.7 as a buffer solution. Fractions containing the target protein were pooled and concentrated by Amicon Ultra 10 kDa, reaching a volume of 3.5 mL. Subsequently, the protein solution was loaded onto a Hi Load Superdex 16/600 200 pg column for Size Exclusion Chromatography (SEC). Isocratic elution was performed with 1.2 column volumes of 10 mM HEPES, 300 mM NaCl, 1 mM EDTA and 1 mM DTT pH 7.5 as a buffer solution. The final protein yield resulted in 45 mg/L of culture. The purity of CVB3 3C^Pro^ was checked by SDS-PAGE analysis. The production of ^15^N-labeled and ^13^C,^15^N-labeled CVB3 3C^Pro^ was performed by following the same protocol, just switching to a standard M9-medium containing (^15^NH_4_)_2_SO_4_ (1.2 g/L) and ^13^C-glucose (4 g/L) as sources of nitrogen and carbon, respectively.

### 2.2. Analytical Gel Filtration

The protein size of CVB3 3C^Pro^ was analyzed using analytical gel filtration (Superdex 200 10/300 increase column; Cyativa, MA, USA) calibrated with gel filtration marker calibration kit, 6500–66,000 Da. Purified samples in 25 mM MES and 150 mM NaCl at pH 6.5 as a buffer was loaded on the column pre-equilibrated with 25 mM MES and 150 mM NaCl at pH 6.5 as a running buffer. Elution profiles were recorded at 280 nm with a flow rate of 0.75 mL/min. A standard curve of the logarithm of the molecular weight of the standards vs. K_av_ = (V_e_ − V_0_)/(V_t_ − V_0_) (V_e_, elution volume; V_0_, dead volume; V_t_, total volume) was used to calculate the apparent molecular mass of CVB3 3C^Pro^.

### 2.3. Crystallization, Data Collection and Structure Solution

Crystals of CVB3 3C^Pro^ in complex with the GC-376 PROTAC precursor were obtained through co-crystallization in a sitting drop by adding a 2 µL aliquot of reservoir buffer (0.1 M Tris-HCl, 0.2 M MgCl_2_ and 26% PEG4000, pH 8.5) to 2 µL of protein solution (20 mM Tris-HCl, 150 mM NaCl, 1 mM EDTA and 1 mM DTT, pH 7.8) containing the ligand (2–3-fold concentration with respect to the protein and dissolved in 10% DMSO); trays were stored at 20 °C. The protein concentration in the sample was 5 mg/mL.

The dataset was collected in-house, using a BRUKER D8 Venture diffractometer equipped with a PHOTON III detector, at 100 K; the crystals used for data collection were cryo-cooled using 25% ethylene glycol in the mother liquor. The GC-376 PROTAC precursor–protein adduct crystal was refined at 1.9 Å resolution: it belongs to space group C2 with one molecule in the asymmetric unit, a solvent content of about 50% and a mosaicity of 0.4–0.6°. The data were processed using the program XDS [51] and were reduced and scaled using XSCALE [51], and amplitudes were calculated using XSCALE [51]. The structure was solved with the molecular replacement technique using the 7QUW structure as the template model. The successful orientation and translation of the molecule within the crystallographic unit cell was determined with MOLREP [52]. The refinement and water molecule fitting was carried out using PHENIX [53], applying default TLS restraints. In between the refinement cycles, the model was subjected to manual rebuilding using COOT [54]. The quality of the refined structures was assessed using the program MOLPROBITY [55]. Data processing and refinement statistics for the GC-376 PROTAC precursor–protein adduct are shown in Table S1. The coordinates and structure factors have been deposited in the PDB under the accession code 8S6F. The software used for structural visualization is PyMOL (Schrodinger, LLC (2015), The PyMOL Molecular Graphics System, Version 1.8).

### 2.4. NMR Spectroscopy

Conventional multidimensional NMR techniques based on 3D triple-resonance experiments [56] were performed on an AVANCE 500 spectrometer equipped with a cryogenically cooled probe to obtain a backbone resonance assignment of CVB3 3C^Pro^ with and without the GC-376 PROTAC precursor at 308 K. The used NMR sample condition was 50 mM phosphate buffer at pH 6.0 containing 100 mM NaCl, 50 mM arginine, 50 mM glutamate, 1 mM DTT and 1 mM EDTA and 10% (*v*/*v*) D_2_O. All the NMR spectra were processed using the standard software Bruker: Topspin 4.2 and analyzed with the program CARA [57].

In order to monitor the interaction of CVB3 3C^Pro^ with the GC-376 PROTAC and its precursor, we performed an NMR titration, based on the acquisition of ^1^H-^15^N HSQC experiments at 308 K on a Bruker AVANCE 950 MHz, by adding aliquots (up to a maximum of 10 μL of final added volume) of the small molecule (GC-376 PROTAC or its precursor) dissolved in D3-acetonitrile to the NMR tube containing ^15^N-labeled CVB3 3C^Pro^ (0.2–0.3 mM) up to a 1:1 ratio. All NMR titration data were analyzed comparing the ^1^H-^15^N HSQC spectra recorded along the additions of the small molecule with that of the initial state as well as with ^1^H-^15^N HSQC blank spectra of ^15^N-labeled CVB3 3C^Pro^ recorded from titrating the protein with the same aliquot of D3-acetonitrile, in order to identify the chemical shift changes induced by the organic solvent. In such a way, following the chemical shift changes observed in the ^1^H-^15^N HSQC maps along each stepwise titration, we were able to selectively assign the residues affected by protein–protein interactions. Signals showing chemical shift changes occurring on a slow exchange regime of the NMR time scale were considered when mapping the interaction surface on CVB3 3C^Pro^ upon binding with the small molecule. The observed chemical shift changes were calculated as backbone-weighted average chemical shift differences, i.e., Δδ_avg_(HN), i.e., (((ΔH)^2^ + (ΔN/5)^2^)/2)^1/2^, where ΔH and ΔN are chemical shift differences for backbone amide ^1^H and ^15^N nuclei, respectively. The estimation of the chemical shift threshold value for defining meaningful chemical shift differences was obtained by averaging Δδ_avg_(HN) values plus one standard deviation (1σ), following the standard procedure used in NMR protein–protein interaction studies [58].

The backbone dynamic properties of CVB3 3C^Pro^ have been sampled through ^15^N relaxation measurements. NMR experiments for measuring ^15^N longitudinal (R_1_) and transverse (R_2_) relaxation rates [59] and [^1^H]^15^N heteronuclear NOE values [60] were recorded at 298 K at 500 MHz, using a protein concentration of 300 μM. A temperature dependence of the backbone NH chemical shifts in the ^1^H-^15^N HSQC spectra allowed us to obtain the backbone resonance assignment of CVB3 3C^Pro^ at 298 K. ^15^N R_1_, ^15^N R_2_ and steady-state [^1^H]^15^N NOEs were obtained with previously described pulse sequences, which employ gradient selection and sensitivity enhancement, as well as minimal water suppression. ^15^N R_2_ were measured with a refocusing time (τ_CPMG_) of 450 μs with the Carr–Purcell–Meiboom–Gill (CPMG) sequence. In all experiments, the water signal was suppressed with the “water flipback” scheme. ^15^N R_1_ and ^15^N R_2_ relaxation rates were obtained by fitting the cross-peak volumes (I), measured as a function of the relaxation delay, to a single-exponential decay as described in the literature [61]. Heteronuclear [^1^H]^15^N NOE values were calculated as the ratio of peak volumes in spectra recorded with and without saturation. The analysis of the uncertainties of ^15^N R_1_ and ^15^N R_2_ relaxation rates was carried out by comparing the peak intensity on duplicated spectra having the same relaxation delay. Estimates of the molecular tumbling value under the chosen experimental conditions of magnetic field and temperature were obtained using the program HydroNMR following the standard procedure [62].

### 2.5. Enzyme Inhibition Kinetics Assay

The CVB3 3C^Pro^ enzyme inhibition activity of the synthesized GC-376 PROTAC and its precursor were analyzed through a fluorometric assay using the fluorogenic substrate Hilyte Fluor-488-ESATLQSGLRKAK-(QXL-520)-NH_2_ (Anaspec). All the measurements were performed in 96-well plates with a Fluostar Optima microplate reader (BMG Labtech, Ortenberg, Germany) analogously to what has previously been performed [12]. Excitation and emission wavelengths were 490 and 520 nm, respectively. All incubations were performed at 30 °C in 10 mM HEPES buffer containing 150 mL NaCl, 1 mM EDTA and 1mM DTT at pH = 7.4. The GC-376 PROTAC or the GC-376 PROTAC precursor were preincubated with the CVB3 3C^Pro^ enzyme (59 nM) for 10 min at 30 °C before the reaction was started by the addition of the fluorogenic substrate (1 μM). The increase in fluorescence signal was monitored over 30 min (λ_ex_ = 490 nm, λ_em_ = 520 nm) at 30 °C. The percentages of inhibition for the tested molecules were determined through the equation (1 − V_s_/V_o_) × 100, where vs. is the initial velocity in the presence of the inhibitor and V_o_ is the initial velocity of the uninhibited reaction. The initial velocity was calculated, using the data analysis software MARS 2875A embedded in the Fluostar Optima instrument, as the slope of the linear regression of the curves in the initial range (0–5 min). The IC_50_ values were obtained by dose–response measurements using an inhibitor range of concentrations from 0.1 nM to 0.3 mM for the GC-376 PROTAC and 0.01 nM to 0.1 mM for the GC-376 PROTAC precursor. All the experiments were performed in triplicate, and the data collected were analyzed using GraphPad 5.0 Software Package (GraphPad Prism, Inc., San Diego, CA, USA). The percentages of inhibition at each inhibitor concentration were fitted using the non-linear fitting function ‘log(inhibitor) vs. response’ implemented in GraphPad.

## 3. Results

### 3.1. Crystal Structure of CVB3 3C^Pro^ in Complex with GC-376 PROTAC Precursor

To characterize the interaction of CVB3 3C^Pro^ with both the GC-376 PROTAC and its precursor, the same compounds previously used by us with SARS-CoV-2 3CL^Pro^ (Figure 1B), we performed crystallization trials through both ligand-co-crystallization and soaking strategies. Well-diffracting crystals were obtained only in the case of the GC-376 PROTAC precursor. This result differs from what was obtained with SARS-CoV-2 3CL^Pro^, whose well-diffracting crystals were obtained with both the GC-376 PROTAC precursor and the GC-376 PROTAC by soaking and co-crystallization, respectively. The crystal structure of CVB3 3C^Pro^ in complex with the GC-376 PROTAC precursor was solved at 1.9 Å resolution, which provided atomic details of the CVB3 3C^Pro^–ligand interactions. The overall 3D structure clearly resembles that of the free protein (PDB IDs 2VB0 and 3ZYD) with RMSD computed on C-alpha atoms of 0.55 and 0.33 Å, respectively. The structure of CVB3-3C^pro^ adopts a chymotrypsin protein fold. The N-terminus starts with an α-helix of residues 1–14 and is followed by two topologically equivalent β-barrels comprising residues 15–77 and 100–173, which pack together to form a narrow groove for substrate binding. The catalytic triad of Cys147, His40 and Glu71 is located in the cleft between the two β-barrels.

**Figure 2:**
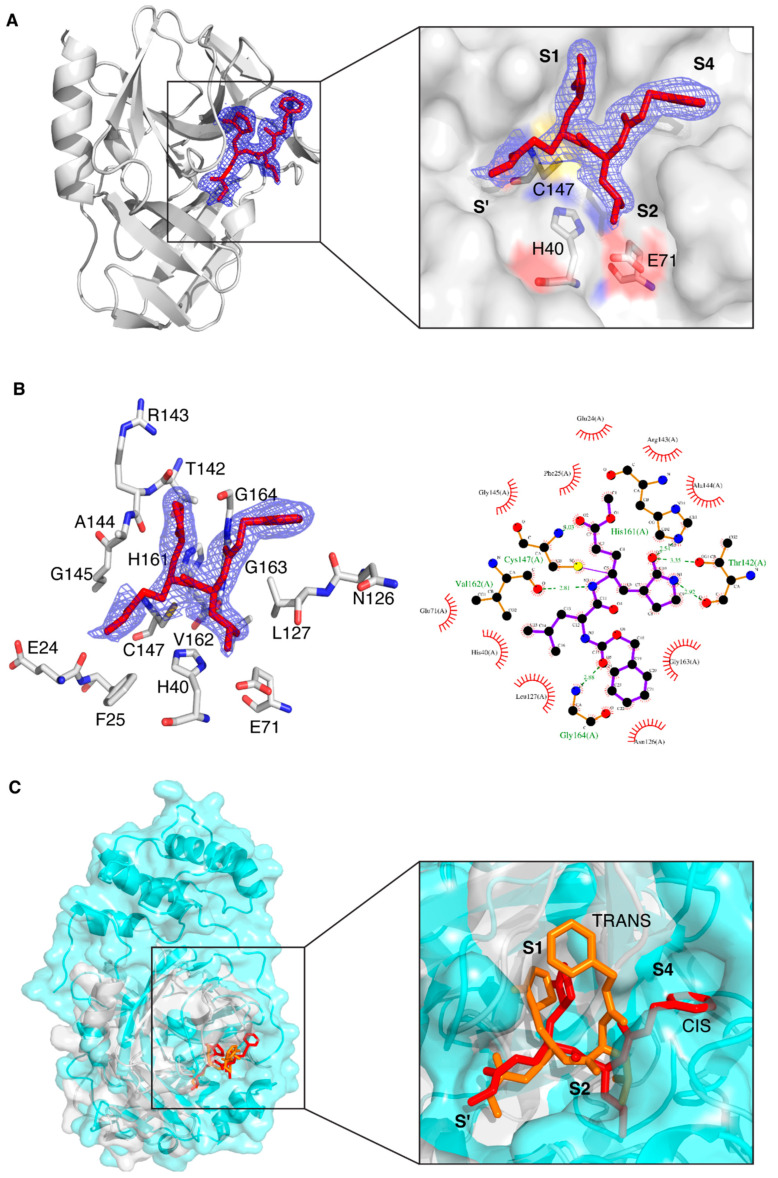
Crystal structure of CVB3 3C^Pro^ in complex with the GC-376 PROTAC precursor. (**A**) Left: Ribbon diagram of CVB3 3C^Pro^ bound to the GC-376 PROTAC precursor, with the 2Fo-Fc electron density map (blue mesh) contoured around the ligand at 1.0σ. Right: Close-up view of the binding site, highlighting the interaction of the GC-376 PROTAC precursor with key catalytic residues (Cys147, His40 and Glu71) in the active site. The subsites are also indicated. (**B**) Left: Representation of the binding interactions between the GC-376 PROTAC precursor and the surrounding residues of CVB3 3C^Pro^, with the 2Fo-Fc electron density map as above. Right: Schematic 2D Ligplot representation showing the detailed binding interactions and hydrogen bonds between the ligand and active site residues, with hydrophobic interactions depicted as red arcs. (**C**) Left: Structural superimposition of CVB3 3C^Pro^ (light gray, PDB ID 8S6F) and SARS-CoV-2 3CL^Pro^ (cyan, PDB ID 8OKB) in complex with the GC-376 PROTAC precursor. The latter is shown as red sticks and orange sticks for CVB3 3C^Pro^ and SARS-CoV-2 3CL^Pro^, respectively. Right: Close-up of the binding pocket, showing the analogous orientation of the precursor in both active sites. The different conformations of the benzyloxycarbonyl group are also visible: “cis” for CVB3 3C^Pro^ and “trans” for SARS-CoV-2 3CL^Pro^.

Concerning the presence of the protease ligand, we observed that the electron density is well defined for all atoms (Figure 2A). The ligand has been refined at full occupancy and the B-factor values of the atoms that are visible in the electron density are in line with those of the neighboring protein atoms. The most relevant feature is the binding mode of the ligand to the catalytic Cys147, which occurs via a covalent bond between C5 and the sulfur atom of Cys147. From a crystallographic point of view, this is supported by the fact that the bond length spontaneously refines to values around 1.8 Å, which is coherent with a C-S covalent bond. From a chemical point of view, it is known that the formation of a covalent (even reversible) bond between the unsaturated carbon and the sulfur atom is very likely [63,64]. Besides the covalent bond, other polar and non-polar interactions keep the protease ligand in place (Figure 2B). The protein atoms involved in direct hydrogen bond interactions with the ligand are, namely, the backbone oxygen of Thr142, one of the imidazole nitrogen atoms of His161, the backbone oxygen of Val162, the backbone nitrogen atom of Gly164 and the backbone nitrogen atom of Cys147. Several more residues are involved in hydrophobic interactions, and this is in agreement with the relatively non-polar nature of the ligand.

Despite the structural difference in the subsites between SARS-CoV-2 3CL^Pro^ and CVB3 3C^Pro^ (shown in Figure 1A), the cavity of CVB3 3C^Pro^ that hosts the ligand has a similar shape with respect to that of SARS-CoV-2 3CL^Pro^ (PDB ID 8OKC) (Figure 2C); for this reason, the binding mode and interactions of the GC-376 PROTAC precursor in CVB3 3C^Pro^ are similar to those in the 8OKB and 8OKC SARS-CoV-2 3CL^Pro^ structures [12]. The only exception is the conformation of the phenyl ring of the benzyloxycarbonyl group, which is basically flipped by 90°. This effect is due to a different structural arrangement of the C-terminal segment of CVB3 3C^Pro^ with respect to that of SARS-CoV-2 3CL^Pro^, which makes the substrate cavity of CVB3 3C^Pro^ more open. The larger available volume allows the phenyl ring to rotate in a position that is structurally more congested in the structure of SARS-CoV-2 3CL^Pro^ (Figure 2C). This evidence also suggests that the ligand is versatile and is able to efficiently interact with different protein targets, provided, of course, that there is a certain degree of structural similarity among them. The alpha-helical region capping from the top the substrate cavity in SARS-CoV-2 3CL^Pro^, which is absent in the CVB3 3C^Pro^, does not determine a different orientation of the nearby Leu side chain of the ligand in the two structures. The ligand-binding site of CVB3 3C^Pro^ has also been superposed to that of CVB3 3C^Pro^ bound to a ligand similarly to the GC-376 PROTAC precursor (PDB ID 5NFS); in this case, the overall positions of the ligands are superposable (Figure S1).

All these results support that an antiviral PROTAC molecule acting on both coronaviral and enteroviral proteases can be constructed starting from our dipeptidyl protease ligand.

### 3.2. Structural Characterization of CVB3 3C^Pro^ by Solution NMR

**Figure 3:**
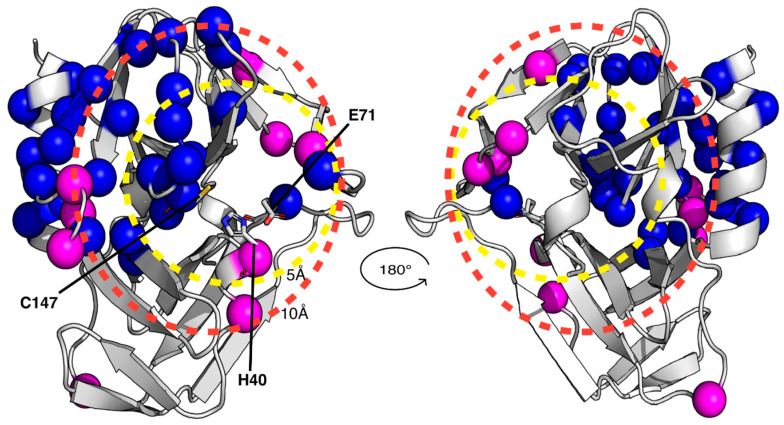
Structural and dynamic characterization of CVB3 3C^Pro^ by solution NMR at 308 K. Backbone amide protons that are broadened beyond detection and those exhibiting elevated R_2_/R_1_ relaxation rates or negative ^1^H-^15^N NOE values are highlighted as blue and magenta spheres, respectively, on the crystal structure of CVB3 3C^Pro^. Dashed circles indicate spatial distances of 5 Å and 10 Å from the active site, demarcating regions surrounding the binding pocket. The catalytic triad is also indicated as sticks.

Because of the failure to obtain crystals of the GC-376 PROTAC-CVB3 3C^Pro^ complex, we decided to apply a solution NMR strategy to characterize the interaction between the GC-376 PROTAC and CVB3 3C^Pro^. Since the backbone resonance assignment of CVB3 3C^Pro^ was not available, the first step was to find the experimental sample conditions suitable for obtaining high-quality 3D triple-resonance experiments required to extract the sequential information. The protein was produced in a ^15^N- and ^13^C-labeled medium and purified to a purity > 95% with two purification steps (Figure S2). Analytical gel filtration showed that the protein is homogenous with just one species present in solution corresponding to the monomeric form (Figure S2). The ^1^H-^15^N HSQC spectrum of CVB3 3C^Pro^ at 298 K in 50 mM phosphate buffer with 100 mM NaCl at pH 6.0 shows well-dispersed amide signals with few peaks clustered in the random-coil region (Figure S3). Nevertheless, the high quality of the ^1^H-^15^N HSQC spectrum is not reflected in 3D triple-resonance NMR experiments needed to achieve a complete backbone resonance assignment (Figure S3). Indeed, several NHs in the 3D triple-resonance NMR experiments lack the sequential pattern of signals required to unambiguously perform backbone resonance assignment. Moreover, in these conditions, the protein has a strong tendency to precipitate over time in the NMR tube. Thus, to find suitable conditions to accomplish complete backbone resonance assignment, we performed a temperature dependence NMR analysis at different pHs and ionic strengths. As a result, we were able to improve the quality of the 3D triple-resonance experiments by using a 50 mM phosphate buffer at pH 6.0 containing 100 mM NaCl, 50 mM arginine, 50 mM glutamate, 1 mM DTT and 1 mM EDTA [65] and by acquiring the NMR spectra at 308 K. In these experimental conditions, the backbone amide NMR signals significantly narrowed, and the number of Cα and Cβ resonances observed in the 3D spectra increased significantly. We were thus able to assign about 80% of the backbone NHs of CVB3 3C^Pro^. In particular, we assigned all the cross-peaks detected in the ^1^H-^15^N HSQC spectrum, indicating that the unassigned backbone NHs are missing since they are very weak or broadened beyond detection (Table S2). Mapping the latter NHs on the structure of CVB3 3C^Pro^, we observed that they are located in a large area involving the Cys···His···Glu residues of the catalytic triad, the substrate-binding subsites, part of the β-strand in contact with subsite S1 and the proximal N-terminal helix (Figure 3). This finding suggests that conformational motions on the μs-ms time scale occur in this region. To further investigate this aspect, we have measured ^15^N longitudinal (R_1_) and transverse (R_2_) relaxation rates and [^1^H]^15^N heteronuclear NOE values of CVB3 3C^Pro^ (Figure S4). These data were used to obtain insights both into the motions of the backbone NHs at different time scales and to calculate, from the R_2_/R_1_ ratio, the overall correlation time for molecular tumbling (τ_m_). In agreement with the results of the analytical gel filtration, the experimentally calculated τ_m_ value is 13.8 ± 1.9 ns, as expected for a protein of this size in a monomeric state and perfectly matching the τ_m_ value estimated by HYDRONMR [62] (13.7 ns) based on the monomeric crystal structure of CVB3 3C^Pro^ (PDB ID 3ZYD). The residues showing negative [^1^H]^15^N NOE values (Lys42, Ser107, Phe109 and Thr132), indicating backbone motions on the ps-ns time scale, as well as those showing high R_2_ over R_1_ ratios (His40, Asn80, Asn105, Glu121, Leu127, Thr132 and Arg134), indicating backbone motions on the μs-ms time scale, were mapped on the structure of CVB3 3C^Pro^ (Figure 3). These NHs are again located in the area involving the catalytic triad, the substrate-binding subsites and some residues of the loop close to subsite S1. We can thus conclude from both the broadening-beyond-detection effects as well as from high internal flexibility in the ps time scale, that the backbone of a large protein region, (i.e., the catalytic triad, the substrate-binding subsites, the β-strand in contact with subsite S1 and the proximal N-terminal helix), is highly dynamic, thus preventing a complete backbone resonance assignment. However, these findings suggested to us that the addition of a ligand could dampen the highly dynamic properties of the ligand-binding region. To test this hypothesis, we have added one equivalent of the GC-376 PROTAC precursor to CVB3 3C^Pro^ and recorded a ^1^H-^15^N HSQC spectrum (Figure S3) and a full set of the 3D triple-resonance experiments. The results show that the quality of the 3D triple-resonance experiments is significantly improved (Figure S3), in such a way allowing us to obtain a complete and accurate backbone resonance assignment at 308 K for the GC-376 PROTAC precursor-bound state of CVB3 3C^Pro^ (97% of assigned backbone NHs), with only five missing NHs (Table S2). The backbone resonance assignment at 308 K has been deposited in the BioMagResBank (BMRB ID 52547).

The observed quench of dynamics of a large part of the protein backbone indicates that the cavity hosting the substrate samples different backbone conformations in the absence of the substrate. This structural feature allows a selection of the conformation necessary to covalently bind the substrate, which is the chemical step fundamentally required to functionally activate the enzymatic reaction.

### 3.3. Mapping the Interaction of the GC-376 PROTAC and Its Precursor with CVB3 3C^Pro^ by Solution NMR

**Figure 4:**
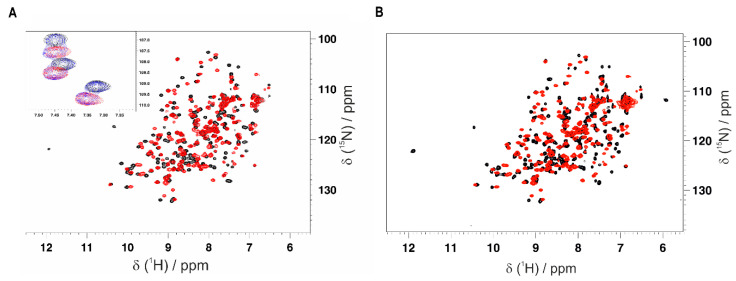
Monitoring the interaction of the GC-376 PROTAC and its precursor with CVB3 3C^Pro^ by solution NMR. Overlay of the ^1^H-^15^N HSQC spectra of CVB3 3C^Pro^ before (red) and after (black) the addition of 1 equivalent of the GC-376 PROTAC precursor (**A**) and GC-376 PROTAC (**B**) recorded at 308K. In the inset of panel A, three NH signals in a slow exchange regime of the NMR time scale are shown at 0 (red), 0.5 (blue) and 1 (black) equivalent of the GC-376 PROTAC precursor additions.

**Figure 5:**
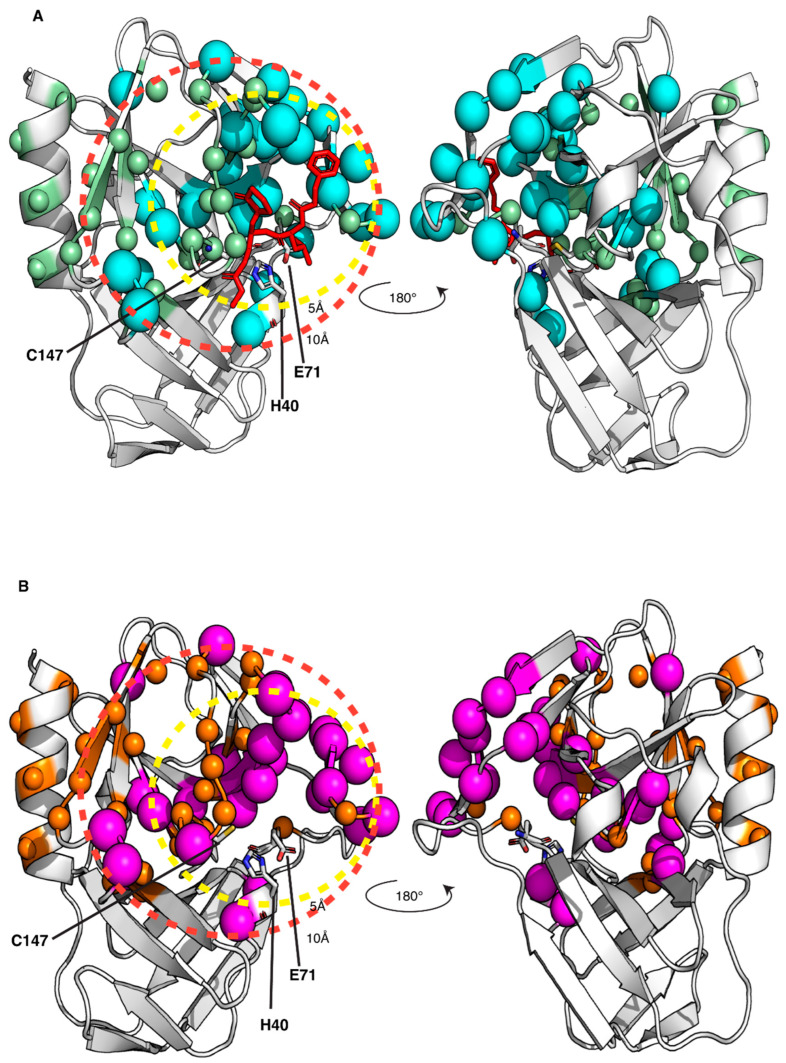
Mapping the interaction of the GC-376 PROTAC and its precursor with CVB3 3C^Pro^ onto the crystal structure of the protein bound to the GC-376 PROTAC precursor (PDB ID 8S6F). The backbone NHs showing chemical shifts changes larger than the threshold value, as observed in Figure S5A,B, are mapped as cyan and magenta spheres for the GC-376 PROTAC precursor (**A**) and GC-376 PROTAC (**B**) titrations, respectively. The backbone NHs, unassigned in the CVB3 3C^Pro^ and displaying increasing intensities along the stepwise additions of the GC-376 PROTAC precursor (light-green spheres) and of the GC-376 PROTAC (orange spheres) are also shown in panels (**A**) and (**B**) respectively. The dashed circles indicate spatial distances of 5 Å and 10 Å from the active site, demarcating regions surrounding the binding pocket. The catalytic triad is shown as sticks.

Thanks to having obtained the backbone resonance assignment of CVB3 3C^Pro^ with and without the GC-376 PROTAC precursor, we can identify the residues of CVB3 3C^Pro^ whose NH resonances are affected by the binding of the GC-376 PROTAC precursor as well as of the GC-376 PROTAC. An NMR titration was first performed by adding the GC-376 PROTAC precursor stepwise to ^15^N-labeled CVB3 3C^Pro^. Several NH signals are affected by the GC-376 PROTAC precursor additions, showing a slow exchange regime on the NMR time scale (Figure 4A and Figure S5A, Table S3). Moreover, new NH signals appear with increasing intensities along the stepwise additions of the GC-376 PROTAC precursor with their chemical shifts corresponding to those of the GC-376 PROTAC precursor-bound species (Figure S5A, Table S4). Taken together, these findings indicate that the GC-376 PROTAC precursor is tightly bound to CVB3 3C^Pro^. Mapping both these types of NH changes on the crystal structure of CVB3 3C^Pro^ in complex with the GC-376 PROTAC precursor (PDBID 8S6F) (Figure 5A), we can observe that the large majority of the backbone NHs surround the ligand but also include the β-strand in contact with subsite S1 and the proximal N-terminal helix. These results indicate the specific binding of the GC-376 PROTAC precursor to the cavity containing the Cys···His···Glu/Asp catalytic triad. We then used the same approach to investigate the binding of the GC-376 PROTAC: an NMR titration was thus performed by adding the GC-376 PROTAC stepwise to ^15^N-labeled CVB3 3C^Pro^. The observed chemical shift changes similarly reproduce those occurring upon the GC-376 PROTAC precursor additions, with signals displaying a slow exchange regime on the NMR time scale and new signals appearing with increasing intensities along the titration (Figure 4B and Figure S5B, Tables S3 and S4). The residues whose chemical shifts change upon the addition of PROTAC were mapped on the crystal structure of 3C^Pro^ bound the PROTAC precursor (PDBID 8S6F) (Figure 5B). Upon interaction with 3C^Pro^, PROTAC affects the same regions of its precursor. This is also confirmed by comparing the effects on the backbone chemical shifts of the binding of the GC-376 PROTAC to ^15^N-labeled CVB3 3C^Pro^ with respect to those generated by the GC-376 PROTAC precursor (Figure S6). Indeed, the obtained ΔΔδ_avg_ values are all below a threshold of ±0.05 ppm with the exception of four residues (Leu37, Asn69, Leu127 and Gly129) [58]. However, the latter four discrepancies do not indicate a meaningful difference in binding between PROTAC and its precursor. This is because the chemical shift differences in Leu37 and Asn69 are not meaningful in both NMR titrations with the GC-376 PROTAC and its precursor (i.e., below the threshold of 0.14 ppm; see Figure S5). Furthermore, the chemical shift differences in Leu127 and Gly129, although above the threshold in both titrations, show a difference as little as 0.054–0.062 ppm (Figure S5). Only two small differences over 175 backbone NHs indicate that the two molecules interact with CVB3 3C^Pro^ in a very similar mode. Thus, we can conclude that the linker and the pomalidomide ligand moiety are not tightly interacting with the protein surface, thus displaying a high degree of mobility. These data are coherent with our previous findings concerning the binding of the same GC-376 PROTAC molecule to SARS-CoV-2 3CL^Pro^ [12].

### 3.4. Enzyme Inhibition Kinetics of GC-376-PROTAC and Its Precursor

**Figure 6:**
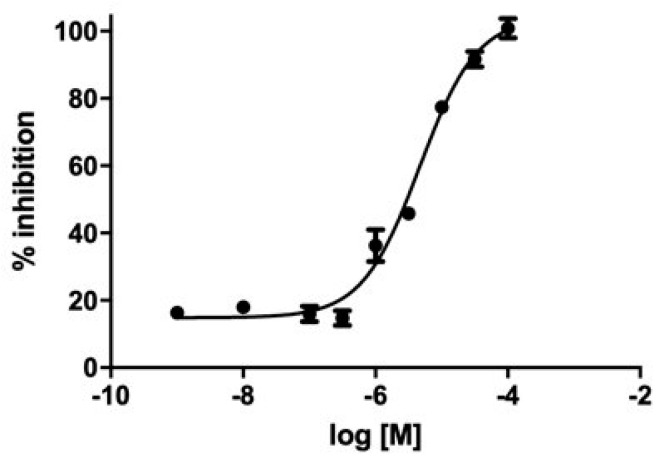
Inhibition curve of the GC-376 PROTAC precursor. M in the log refers to molar concentration.

We evaluated GC-376-PROTAC and its precursor for their ability to inhibit CVB3 3C^Pro^ through a fluorimetric enzyme inhibition kinetics assay using Hilyte Fluor-488-ESATLQSGLRKAK-(QXL-520)-NH_2_ as the substrate. CVB3 3C^Pro^ and EV71 3C (PDB ID 5BPE) are two viral proteases with a very high sequence identity (98.4%) and whose structures are fully superimposable. EV71 3C as well as SARS-CoV-2 3CL^Pro^ are known to cleave several substrates in the dipeptide sequence consisting of Gln followed by a small amino acid (Ser or Gly), like in the substrate TSAVLQSGFRKM, used by several authors in the literature [48,49,66], and in the substrate used in this work containing the same LQSG epitope. These findings support that the peptide used here is also a suitable substrate for CVB3 3C^Pro^. Both ligands showed an inhibition activity in the micromolar range. Specifically, the GC-376 PROTAC showed 32 ± 3% inhibition at 100 μM, and the GC-376 PROTAC precursor showed an IC_50_ of 4.6 μM (pIC_50_ = 5.28 ± 0.07) (Figure 6). Such values are in line with that displayed by SARS-CoV-2 3CL^Pro^ (1.35 μM) [12], still supporting similar molecular recognition in the active site of both enzymes. The 4.6 μM value of the GC-376 PROTAC precursor is also comparable to those of compounds structurally similar to the GC-376 PROTAC precursor and inhibiting CVB3 3C^Pro^ [48].

## 4. Discussion

**Figure 7:**
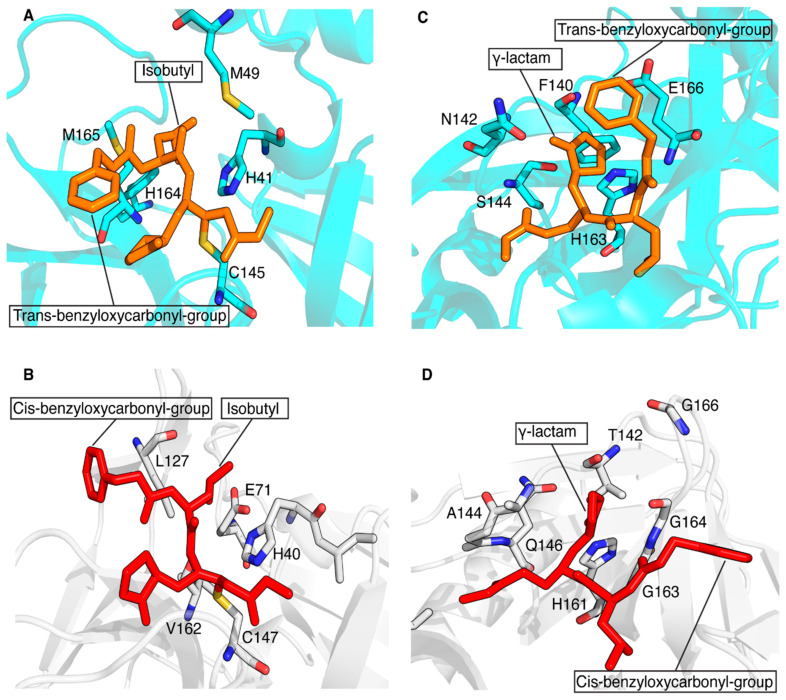
Structural comparison of the ligand–protein interactions in CVB3 3C^Pro^ (PDB ID 8S6F) and SARS-CoV-2 3CL^Pro^ (PDB ID 8OKB). The side chains of the residues surrounding the isobutyl group (**A**) and γ-lactam ring (**C**) of the GC-376 PROTAC precursor (in orange) are shown as sticks on the SARS-CoV-2 3CL^Pro^ structure. The trans-conformation of benzyloxycarbonyl group is indicated. The side chains of the residues surrounding the isobutyl group (**B**) and the γ-lactam ring (**D**) of the GC-376 PROTAC precursor (in red) are shown as sticks on the CVB3 3C^Pro^ structure. The cis-conformation of the benzyloxycarbonyl group is indicated. The key residues are shown as sticks.

The conservation of the main protease in viral genomes and the fact that humans do not have a homologous protease make this enzyme family an ideal target for developing antiviral drugs with minimized toxicity against the host cell. These features have driven the development of several classes of viral inhibitors able to block the function of this target. Among them, GC-376 is one of the most efficient 3CL protease inhibitors found to inhibit the activity of coronavirus 3CL^Pro^ [67]. A recent study showed that GC-376 is a potent 3CL protease inhibitor that binds with different kinds of coronavirus 3CL^Pro^ and a variety of 3CL^Pro^ mutants of SARS-CoV-2 to exert inhibitory activity [68]. In particular, GC-376 was shown to have a strong inhibitory activity against three pathogenic coronaviruses, i.e., SARS-CoV, MERS-CoV and SARS-CoV-2 3CL^Pro^. Recently, we have shown that a GC-376-based peptidomimetic PROTAC specifically targets and degrades the dimeric SARS-CoV-2 3CL^Pro^ protein [12]. Several other PROTACs have been recently described to perform this function against both α- and β-groups of coronaviruses [11,13,14,69]. Here, we have extended the application of our GC-376-based peptidomimetic PROTAC to 3C^Pro^ protease, which is present in some members of the large genus enterovirus. Specifically, we structurally characterized the interaction of the GC-376 PROTAC with CVB3 3C^Pro^, a member of the enteroviral proteases, and we found that the GC-376 PROTAC interacts similarly to what found with SARS-CoV-2 3CL^Pro^. The GC-376 molecule penetrates in the substrate-binding site of CVB3 3C^Pro^, while the linker and the pomalidomide molecule are not tightly bound to CVB3 3C^Pro^ but remain exposed to the solvent. The binding of GC-376 to CVB3 3C^Pro^ is similar to its binding in SARS-CoV-2 3CL^Pro^, but there are some notable differences. The main difference lies in the orientation of the benzyloxycarbonyl group of the GC-376 molecule, which is significantly altered between the two structures. This difference is also observed when the GC-376 inhibitor binds to 3CL^Pro^ of SARS-CoV and MERS-CoV [68]. In these crystal structures, the benzyloxycarbonyl group is indeed not immobilized, but it displays two conformations, “cis” and “trans”, for each independent molecule and not a simultaneous presence of cis and trans in the same molecule. Namely, “cis” indicates that the benzyloxycarbonyl group shifted to Thr190, whereas “trans” indicates the benzyloxycarbonyl group shifted to the 2-pyrrolidone ring. Here, we have adopted the same criterion to identify the two possible conformers. Based on a more visual point of view, it is possible to say that in the “cis”-conformation, the phenyl ring lies on the opposite side with respect to the carbonyl oxygen and, conversely, that in the “trans”-conformation, the phenyl group lies on the same side with respect to the carbonyl oxygen. In Figure 7A,C, the trans-conformation of the benzyloxycarbonyl group of the GC-376 PROTAC precursor bound to 3CL^Pro^ of SARS-CoV-2 can be observed (PDB-ID 8OKB). The same trans-conformation of the benzyloxycarbonyl group is present in MERS-CoV (PDB-ID 8IG6). While a cis-conformation occurs for the benzyloxycarbonyl group of the GC-376 precursor bound to CVB3 3C^Pro^ (Figure 7B,D), as occurs in SARS-CoV (PDB-ID 8IG5). This cis- or trans-conformation can be therefore present in 3C^Pro^/3CL^Pro^ proteases, indicating that the S4 subsite of the substrate cavity is an open space where the benzyloxycarbonyl group of GC-376 can extend to the solvent, and it is relatively flexible due to limited stabilizing forces. Other minor structural differences include the ligand interactions with subsites S1 and S2. The isobutyl group, which inserts into the S2 subsite of both proteases, shows hydrophobic interactions with non-polar amino-acid residues (His41, Met49, His164 and Met165) in the 3CL^Pro^ structure of SARS-CoV-2, stabilizing its binding to the S2 site (Figure 7A). This hydrophobic patch is not fully maintained in the CVB3 3C^Pro^ structure. Indeed, the catalytic Glu71, which is absent in coronaviral 3CL^Pro^s, is located with its negative charge next to the isobutyl group (Figure 7B). Finally, the γ-lactam ring of GC-376, which inserts into the S1 subsite of both proteases, forms similar interactions with the catalytic Cys and His161/163, residues conserved in both 3C^Pro^ and 3CL^Pro^ proteases. However, it also has other different surrounding residues in the two proteases. Specifically, there are several residues located in the same structural positions of the S1 subsite of both proteases that are exchanged: Phe140 → Thr142, Asn142 → Ala144, Ser144 → Gln146 and Glu166 → Gly163,164,166 (Figure 7C,D). Overall, we can conclude that, despite considerable sequence diversity in the substrate-binding sites of 3C^Pro^ and 3CL^Pro^, the GC-376 PROTAC can accommodate well in the binding sites of both proteases, suggesting this system as a valuable starting molecular platform for developing a broad-spectrum antiviral PROTAC. Further data concerning the degradation ability of our PROTAC against CVB3 3C^Pro^ in viral cells will be crucial for verifying the applicability of our molecule.

## Data Availability

Coordinates and structure factors have been deposited at the PDB under the accession code 8S6F for CVB3 3C^Pro^ complexed with the GC-376 PROTAC precursor. The backbone resonance assignment of the GC-376 PROTAC precursor-bound state of CVB3 3C^Pro^ at 308 K has been deposited in the BioMagResBank (BMRB ID 52547).

## Supplementary Materials

The following supporting information can be downloaded at https://www.mdpi.com/article/10.3390/biom14101260/s1. Table S1, data collection and refinement statistics of the crystal structure of CVB3 3C^Pro^ in complex with the GC-376 PROTAC precursor; Table S2, list of the unassigned backbone NHs of CVB3 3C^Pro^ and CVB3 3C^Pro^ in complex with the GC-376 PROTAC precursor; Table S3, list of the residues whose NH chemical shifts change above the threshold upon addition of the GC-376 PROTAC and of its precursor; Table S4, list of the residues unassigned in CVB3 3C^Pro^ and displaying increasing intensities along the stepwise additions of the GC-376 PROTAC and of its precursor; Figure S1, superposition of CVB3 3C^Pro^ bound to the GC-376 PROTAC precursor and a similar ligand; Figure S2, expression/purification steps and protein size of CVB3 3C^Pro^; Figure S3, solution NMR spectra acquired at 500 MHz of CVB3 3C^Pro^ and of CVB3 3C^Pro^ bound to the GC-376 PROTAC precursor ; Figure S4, ^15^N relaxation data of CVB3 3C^Pro^; Figure S5, monitoring the interaction of CVB3 3C^Pro^ with the GC-376 PROTAC precursor and GC-376 PROTAC by solution NMR ; Figure S6, comparing the interaction of CVB3 3C^Pro^ with the GC-376 PROTAC with and its precursor CVB3 3CPro by solution NMR; Figure S7, representative kinetic curves of a control experiment in comparison with an inhibition experiment; Figures S8–S11, raw data of experimental and calculated inhibition curves of the GC-376 PROTAC and its precursor.

## References

[1] Komander D., Rape M. (2012). The ubiquitin code. Annu. Rev. Biochem..

[2] Zheng N., Shabek N. (2017). Ubiquitin Ligases: Structure, Function, and Regulation. Annu. Rev. Biochem..

[3] Morreale F.E., Walden H. (2016). Types of Ubiquitin Ligases. Cell.

[4] Deshaies R.J., Joazeiro C.A. (2009). RING domain E3 ubiquitin ligases. Annu. Rev. Biochem..

[5] Schulman B.A., Harper J.W. (2009). Ubiquitin-like protein activation by E1 enzymes: The apex for downstream signalling pathways. Nat. Rev. Mol. Cell Biol..

[6] Ye Y., Rape M. (2009). Building ubiquitin chains: E2 enzymes at work. Nat. Rev. Mol. Cell Biol..

[7] Finley D. (2009). Recognition and processing of ubiquitin-protein conjugates by the proteasome. Annu. Rev. Biochem..

[8] Li K., Crews C.M. (2022). PROTACs: Past, present and future. Chem. Soc. Rev..

[9] Ciulli A.H.O., Jones M.K.L.H. (2022). PROTAC Degraders Mechanism, Recent Advances, and Future Challenges. Protein Homeostasis in Drug Discovery: A Chemical Biology Perspective.

[10] Toure M., Crews C.M. (2016). Small-Molecule PROTACS: New Approaches to Protein Degradation. Angew. Chem. Int. Ed. Engl..

[11] Alugubelli Y.R., Xiao J., Khatua K., Kumar S., Sun L., Ma Y., Ma X.R., Vulupala V.R., Atla S., Blankenship L.R. (2024). Discovery of First-in-Class PROTAC Degraders of SARS-CoV-2 Main Protease. J. Med. Chem..

[12] Grifagni D., Lenci E., De Santis A., Orsetti A., Barracchia C.G., Tedesco F., Bellini Puglielli R., Lucarelli F., Lauriola A., Assfalg M. (2024). Development of a GC-376 Based Peptidomimetic PROTAC as a Degrader of 3-Chymotrypsin-like Protease of SARS-CoV-2. ACS Med. Chem. Lett..

[13] Desantis J., Bazzacco A., Eleuteri M., Tuci S., Bianconi E., Macchiarulo A., Mercorelli B., Loregian A., Goracci L. (2024). Design, synthesis, and biological evaluation of first-in-class indomethacin-based PROTACs degrading SARS-CoV-2 main protease and with broad-spectrum antiviral activity. Eur. J. Med. Chem..

[14] Sang X., Wang J., Zhou J., Xu Y., An J., Warshel A., Huang Z. (2023). A Chemical Strategy for the Degradation of the Main Protease of SARS-CoV-2 in Cells. J. Am. Chem. Soc..

[15] Ma C., Sacco M.D., Hurst B., Townsend J.A., Hu Y., Szeto T., Zhang X., Tarbet B., Marty M.T., Chen Y. (2020). Boceprevir, GC-376, and calpain inhibitors II, XII inhibit SARS-CoV-2 viral replication by targeting the viral main protease. Cell Res..

[16] Kim Y., Lovell S., Tiew K.C., Mandadapu S.R., Alliston K.R., Battaile K.P., Groutas W.C., Chang K.O. (2012). Broad-spectrum antivirals against 3C or 3C-like proteases of picornaviruses, noroviruses, and coronaviruses. J. Virol..

[17] Ito T. (2024). Protein degraders—From thalidomide to new PROTACs. J. Biochem..

[18] Zhu Y.X., Braggio E., Shi C.X., Bruins L.A., Schmidt J.E., Van Wier S., Chang X.B., Bjorklund C.C., Fonseca R., Bergsagel P.L. (2011). Cereblon expression is required for the antimyeloma activity of lenalidomide and pomalidomide. Blood.

[19] Gandhi A.K., Kang J., Havens C.G., Conklin T., Ning Y., Wu L., Ito T., Ando H., Waldman M.F., Thakurta A. (2014). Immunomodulatory agents lenalidomide and pomalidomide co-stimulate T cells by inducing degradation of T cell repressors Ikaros and Aiolos via modulation of the E3 ubiquitin ligase complex CRL4(CRBN.). Br. J. Haematol..

[20] Girardini M., Maniaci C., Hughes S.J., Testa A., Ciulli A. (2019). Cereblon versus VHL: Hijacking E3 ligases against each other using PROTACs. Bioorg. Med. Chem..

[21] Diehl C.J., Ciulli A. (2022). Discovery of small molecule ligands for the von Hippel-Lindau (VHL) E3 ligase and their use as inhibitors and PROTAC degraders. Chem. Soc. Rev..

[22] Anand K., Ziebuhr J., Wadhwani P., Mesters J.R., Hilgenfeld R. (2003). Coronavirus main proteinase (3CLpro) structure: Basis for design of anti-SARS drugs. Science.

[23] Marjomäki V., Kalander K., Hellman M., Permi P. (2021). Enteroviruses and coronaviruses: Similarities and therapeutic targets. Expert Opin. Ther. Targets.

[24] Wen W., Qi Z., Wang J. (2020). The Function and Mechanism of Enterovirus 71 (EV71) 3C Protease. Curr. Microbiol..

[25] Laitinen O.H., Svedin E., Kapell S., Nurminen A., Hytönen V.P., Flodström-Tullberg M. (2016). Enteroviral proteases: Structure, host interactions and pathogenicity. Rev. Med. Virol..

[26] Tan J., George S., Kusov Y., Perbandt M., Anemüller S., Mesters J.R., Norder H., Coutard B., Lacroix C., Leyssen P. (2013). 3C protease of enterovirus 68: Structure-based design of Michael acceptor inhibitors and their broad-spectrum antiviral effects against picornaviruses. J. Virol..

[27] Nikonov O.S., Chernykh E.S., Garber M.B., Nikonova E.Y. (2017). Enteroviruses: Classification, Diseases They Cause, and Approaches to Development of Antiviral Drugs. Biochem. Biokhimiia.

[28] Maier R., Krebs P., Ludewig B. (2004). Immunopathological basis of virus-induced myocarditis. Clin. Dev. Immunol..

[29] Gauntt C., Huber S. (2003). Coxsackievirus experimental heart diseases. Front. Biosci..

[30] Lugo D., Krogstad P. (2016). Enteroviruses in the early 21st century: New manifestations and challenges. Curr. Opin. Pediatr..

[31] Fan W., McDougal M.B., Schoggins J.W. (2022). Enterovirus 3C Protease Cleaves TRIM7 To Dampen Its Antiviral Activity. J. Virol..

[32] Gorbalenya A.E., Donchenko A.P., Blinov V.M., Koonin E.V. (1989). Cysteine proteases of positive strand RNA viruses and chymotrypsin-like serine proteases. A distinct protein superfamily with a common structural fold. FEBS Lett..

[33] Fan K., Wei P., Feng Q., Chen S., Huang C., Ma L., Lai B., Pei J., Liu Y., Chen J. (2004). Biosynthesis, purification, and substrate specificity of severe acute respiratory syndrome coronavirus 3C-like proteinase. J. Biol. Chem..

[34] Zhang L., Lin D., Kusov Y., Nian Y., Ma Q., Wang J., von Brunn A., Leyssen P., Lanko K., Neyts J. (2020). α-Ketoamides as Broad-Spectrum Inhibitors of Coronavirus and Enterovirus Replication: Structure-Based Design, Synthesis, and Activity Assessment. J. Med. Chem..

[35] Anand K., Palm G.J., Mesters J.R., Siddell S.G., Ziebuhr J., Hilgenfeld R. (2002). Structure of coronavirus main proteinase reveals combination of a chymotrypsin fold with an extra alpha-helical domain. Embo J..

[36] Schechter I., Berger A. (1967). On the size of the active site in proteases. I. Papain. Biochem. Biophys. Res. Commun..

[37] Singh E., Khan R.J., Jha R.K., Amera G.M., Jain M., Singh R.P., Muthukumaran J., Singh A.K. (2020). A comprehensive review on promising anti-viral therapeutic candidates identified against main protease from SARS-CoV-2 through various computational methods. J. Genet. Eng. Biotechnol..

[38] Azevedo P., Camargo P.G., Constant L.E.C., Costa S.D.S., Silva C.S., Rosa A.S., Souza D.D.C., Tucci A.R., Ferreira V.N.S., Oliveira T.K.F. (2024). Statine-based peptidomimetic compounds as inhibitors for SARS-CoV-2 main protease (SARS-CoV-2 Mpro). Sci. Rep..

[39] Hayek-Orduz Y., Vásquez A.F., Villegas-Torres M.F., Caicedo P.A., Achenie L.E.K., González Barrios A.F. (2022). Novel covalent and non-covalent complex-based pharmacophore models of SARS-CoV-2 main protease (M(pro)) elucidated by microsecond MD simulations. Sci. Rep..

[40] Fàbrega-Ferrer M., Herrera-Morandé A., Muriel-Goñi S., Pérez-Saavedra J., Bueno P., Castro V., Garaigorta U., Gastaminza P., Coll M. (2022). Structure and inhibition of SARS-CoV-1 and SARS-CoV-2 main proteases by oral antiviral compound AG7404. Antivir. Res..

[41] Göhl M., Zhang L., El Kilani H., Sun X., Zhang K., Brönstrup M., Hilgenfeld R. (2022). From Repurposing to Redesign: Optimization of Boceprevir to Highly Potent Inhibitors of the SARS-CoV-2 Main Protease. Molecules.

[42] Lockbaum G.J., Henes M., Lee J.M., Timm J., Nalivaika E.A., Thompson P.R., Kurt Yilmaz N., Schiffer C.A. (2021). Pan-3C Protease Inhibitor Rupintrivir Binds SARS-CoV-2 Main Protease in a Unique Binding Mode. Biochemistry.

[43] Dai W., Jochmans D., Xie H., Yang H., Li J., Su H., Chang D., Wang J., Peng J., Zhu L. (2022). Design, Synthesis, and Biological Evaluation of Peptidomimetic Aldehydes as Broad-Spectrum Inhibitors against Enterovirus and SARS-CoV-2. J. Med. Chem..

[44] Ramajayam R., Tan K.P., Liu H.G., Liang P.H. (2010). Synthesis and evaluation of pyrazolone compounds as SARS-coronavirus 3C-like protease inhibitors. Bioorg. Med. Chem..

[45] Ramajayam R., Tan K.P., Liang P.H. (2011). Recent development of 3C and 3CL protease inhibitors for anti-coronavirus and anti-picornavirus drug discovery. Biochem. Soc. Trans..

[46] Mandadapu S.R., Weerawarna P.M., Prior A.M., Uy R.A., Aravapalli S., Alliston K.R., Lushington G.H., Kim Y., Hua D.H., Chang K.O. (2013). Macrocyclic inhibitors of 3C and 3C-like proteases of picornavirus, norovirus, and coronavirus. Bioorg. Med. Chem. Lett..

[47] Kumar V., Shin J.S., Shie J.J., Ku K.B., Kim C., Go Y.Y., Huang K.F., Kim M., Liang P.H. (2017). Identification and evaluation of potent Middle East respiratory syndrome coronavirus (MERS-CoV) 3CL(Pro) inhibitors. Antivir. Res..

[48] Lee C.C., Kuo C.J., Ko T.P., Hsu M.F., Tsui Y.C., Chang S.C., Yang S., Chen S.J., Chen H.C., Hsu M.C. (2009). Structural basis of inhibition specificities of 3C and 3C-like proteases by zinc-coordinating and peptidomimetic compounds. J. Biol. Chem..

[49] Kuo C.J., Liu H.G., Lo Y.K., Seong C.M., Lee K.I., Jung Y.S., Liang P.H. (2009). Individual and common inhibitors of coronavirus and picornavirus main proteases. FEBS Lett..

[50] Fili S., Valmas A., Christopoulou M., Spiliopoulou M., Nikolopoulos N., Lichière J., Logotheti S., Karavassili F., Rosmaraki E., Fitch A. (2016). Coxsackievirus B3 protease 3C: Expression, purification, crystallization and preliminary structural insights. Acta Crystallogr. Sect. F Struct. Biol. Commun..

[51] Kabsch W. (2010). XDS. Acta Crystallogr. D. Biol. Crystallogr..

[52] Vagin A.A., Teplyakov A. (2000). An approach to multi-copy search in molecular replacement. Acta Cryst. D.

[53] Adams P.D., Afonine P.V., Bunkòczi G., Chen V.B., Davis I.W., Echols N., Headd J.J., Hung L.-W., Kapral G.J., Grosse-Kunstleve R.W. (2010). PHENIX: A comprehensive Python-based system for macromolecular structure solution. Acta Crystallogr. D. Biol. Crystallogr..

[54] Emsley P., Lohkamp B., Scott W.G., Cowtan K. (2010). Features and development of Coot. Acta Cryst. D.

[55] Chen V.B., Arendall W.B., Headd J.J., Keedy D.A., Immormino R.M., Kapral G.J., Murray L.W., Richardson J.S., Richardson D.C. (2010). MolProbity: All-atom structure validation for macromolecular crystallography. Acta Cryst. D.

[56] Grzesiek S., Bax A. (1993). Amino acid type determination in the sequential assignment procedure of uniformly 13C/15N-enriched proteins. J. Biomol. NMR.

[57] Keller R. (2004). The Computer Aided Resonance Assignment Tutorial.

[58] Williamson M.P. (2013). Using chemical shift perturbation to characterise ligand binding. Prog. Nucl. Magn. Reson. Spectrosc..

[59] Farrow N.A., Muhandiram R., Singer A.U., Pascal S.M., Kay C.M., Gish G., Shoelson S.E., Pawson T., Forman-Kay J.D., Kay L.E. (1994). Backbone dynamics of a free and phosphopeptide-complexed Src homology 2 domain studied by 15N NMR relaxation. Biochemistry.

[60] Grzesiek S., Bax A. (1993). The Importance of Not Saturating H2o in Protein Nmr—Application to Sensitivity Enhancement and Noe Measurements. J. Am. Chem. Soc..

[61] Mandel M.A., Akke M., Palmer A.G. (1995). Backbone dynamics of *Escherichia coli* ribonuclease HI: Correlations with structure and function in an active enzyme. J. Mol. Biol..

[62] Garcia de la Torre J., Huertas M.L., Carrasco B. (2000). HYDRONMR: Prediction of NMR relaxation of globular proteins from atomic-level structures and hydrodynamic calculations. J. Magn. Reson..

[63] Matthews D.A., Dragovich P.S., Webber S.E., Fuhrman S.A., Patick A.K., Zalman L.S., Hendrickson T.F., Love R.A., Prins T.J., Marakovits J.T. (1999). Structure-assisted design of mechanism-based irreversible inhibitors of human rhinovirus 3C protease with potent antiviral activity against multiple rhinovirus serotypes. Proc. Natl. Acad. Sci. USA.

[64] Johnson T.O., Hua Y., Luu H.T., Brown E.L., Chan F., Chu S.S., Dragovich P.S., Eastman B.W., Ferre R.A., Fuhrman S.A. (2002). Structure-based design of a parallel synthetic array directed toward the discovery of irreversible inhibitors of human rhinovirus 3C protease. J. Med. Chem..

[65] Golovanov A.P., Hautbergue G.M., Wilson S.A., Lian L.Y. (2004). A simple method for improving protein solubility and long-term stability. J. Am. Chem. Soc..

[66] Kuo C.J., Shie J.J., Fang J.M., Yen G.R., Hsu J.T., Liu H.G., Tseng S.N., Chang S.C., Lee C.Y., Shih S.R. (2008). Design, synthesis, and evaluation of 3C protease inhibitors as anti-enterovirus 71 agents. Bioorg. Med. Chem..

[67] Fu L., Ye F., Feng Y., Yu F., Wang Q., Wu Y., Zhao C., Sun H., Huang B., Niu P. (2020). Both Boceprevir and GC376 efficaciously inhibit SARS-CoV-2 by targeting its main protease. Nat. Commun..

[68] Lin C., Zhu Z., Jiang H., Zou X., Zeng X., Wang J., Zeng P., Li W., Zhou X., Zhang J. (2024). Structural Basis for Coronaviral Main Proteases Inhibition by the 3CLpro Inhibitor GC376. J. Mol. Biol..

[69] Cheng S., Feng Y., Li W., Liu T., Lv X., Tong X., Xi G., Ye X., Li X. (2024). Development of novel antivrial agents that induce the degradation of the main protease of human-infecting coronaviruses. Eur. J. Med. Chem..

