# Supplementary material for: A Structural Investigation of the Interaction between a GC-376-Based Peptidomimetic PROTAC and Its Precursor with the Viral Main Protease of Coxsackievirus B3
Source: Biomolecules. 2024 Oct 6;14(10):1260. doi: 10.3390/biom14101260 (PMC11506516; doi:10.3390/biom14101260)
Supplement: Supplementary file 1 [file biomolecules-14-01260-s001.zip › biomolecules-3134294-SI.pdf]

SUPPLEMENTARY MATERIALS

Table S1. Data collection and refinement statistics of the crystal structure of CVB3 $3C^{Pro}$ in complex with GC-376 PROTAC precursor.

	**8S6F**
**Wavelength**	1.541
**Resolution range**	35.13 - 1.932 (2.13 - 1.93)*
**Space group**	C 1 2 1
**Unit cell**	76.92 64.35 38.99 90 115.72 90
**Total reflections**	48285 (10396)
**Unique reflections**	23725 (5260)
**Multiplicity**	2.0 (2.0)
**Completeness (%)**	94.73 (86.18)
**Mean I/sigma(I)**	3.56 (0.95)
**Wilson B-factor**	28.98
**R-merge**	0.1379 (0.7405)
**R-meas**	0.1834 (0.9709)
**R-pim**	0.12 (0.6225)
**CC1/2**	0.981 (0.494)
**CC***	0.995 (0.813)
**Reflections used in refinement**	12206 (2726)
**Reflections used for R-free**	611 (136)
**R-work**	0.2384 (0.3192)
**R-free**	0.2634 (0.3220)
**Number of non-hydrogen atoms**	1496
**macromolecules**	1398
**ligands**	33
**solvent**	65
**Protein residues**	180
**RMS(bonds)**	0.003
**RMS(angles)**	0.69
**Ramachandran favored (%)**	94.94
**Ramachandran allowed (%)**	4.49
**Ramachandran outliers (%)**	0.56
**Rotamer outliers (%)**	2.03
**Clashscore**	5.99
**Average B-factor**	37.25
**macromolecules**	37.21
**ligands**	39.20
**solvent**	37.29

*Statistics for the highest-resolution shell are shown in parentheses.

Table S2. List of the unassigned backbone NHs for CVB3 $3C^{Pro}$ and CVB3 $3C^{Pro}$ in complex with GC-376 PROTAC precursor. Sample conditions: 50 mM phosphate buffer at pH 6.0 containing 100 mM NaCl, 50 mM arginine, 50 mM glutamate, 1 mM DTT and 1 mM EDTA.

	**Unassigned backbone NHs**
**CVB3 $3C^{Pro}$**	A3, E5, V8, K12, T26, M27, L70, L102, I104, T106, N111, M112, Y113, I114, V116, G128, F140, T142, R143, A144, Q146, C147, G148, H161, H167, Q168, F170
**CVB3 $3C^{Pro}$ in complex with GC-376 PROTAC precursor**	M11, K19, K76, G155, L175

Table S3. List of the residues whose NH chemical shifts change above the threshold upon addition of GC-376 PROTAC and of its precursor.

**GC-376 PROTAC**	L28, R39, A41, A100, K108, F109, Y122, G123, F124, L125, N126, L127, G129, T130, T132, R134, M135, N139, G149, V150, I160, V162, G163, G164, N165, G166, S171
**GC-376 PROTAC precursor**	L28, R39, A41, A100, K108, F109, Y122, G123, F124, N126, L127, G129, T130, T132, R134, M135, L136, N139, G149, V150, I160, V162, G163, N165, G166, G169, S171

Table S4. List of the residues unassigned in CVB3 $3C^{Pro}$ and displaying increasing intensities along the stepwise additions of GC-376 PROTAC and of its precursor.

**GC-376 PROTAC**	A3, E5, K12, T26, M27, L70, L102, I104, T106, N111, M112, Y113, I114, V116, G128, F140, T142, R143, A144, Q146, C147, G148, H161, H167, Q168, F170
**GC-376 PROTAC precursor**	A3, E5, K12, T26, M27, L70, L102, I104, T106, N111, M112, Y113, I114, V116, F140, T142, R143, A144, Q146, C147, G148, H161, H167, Q168, F170

Figure S1. Superposition of CVB3 3C^{Pro} bound to PROTAC precursor (PDB ID 8S6F) (green) and to (S)-N-benzyl-3-((S)-2-cinnamamido-3-phenylpropanamido)-2-oxo-4-((S)-2-oxopyrrolidin-3-yl)butanamide (PDB-ID 5NFS) (cyan)..

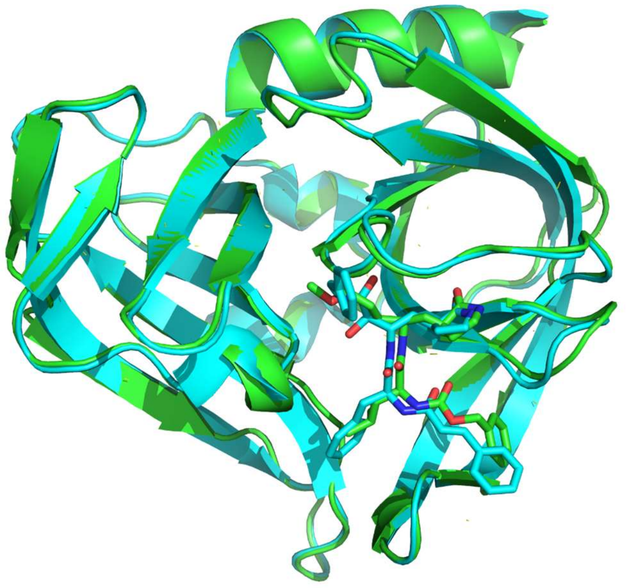

Figure S2. Expression/**purification steps and protein size of CVB3 3C^{Pro}**. (**A**) Analytical gel filtration of CVB3 3C^{Pro} compared with protein standard markers (30 kDa in light blue Carbonic anhydrase (CA) and 8.5 kDa in orange Ubiquitin UB). The theoretical molecular weight of monomeric CVB3 3C^{Pro} is 21 kDa. The protein eluted at a volume corresponding to a molecular mass of 22.9 kDa. (**B**) Analysis of CVB3 3C^{Pro} expression and purification on a Coomassie Blue-stained SDS-PAGE gel. Lanes from the left: molecular marker (labelled in kDa); insoluble and supernatant CVB3 3C^{Pro} expressing cells after overnight expression; Immobilized Metal Affinity Chromatography (IMAC) elution and flow-through; Size-Exclusion Chromatography (SEC) elution of the fractions containing the protein.

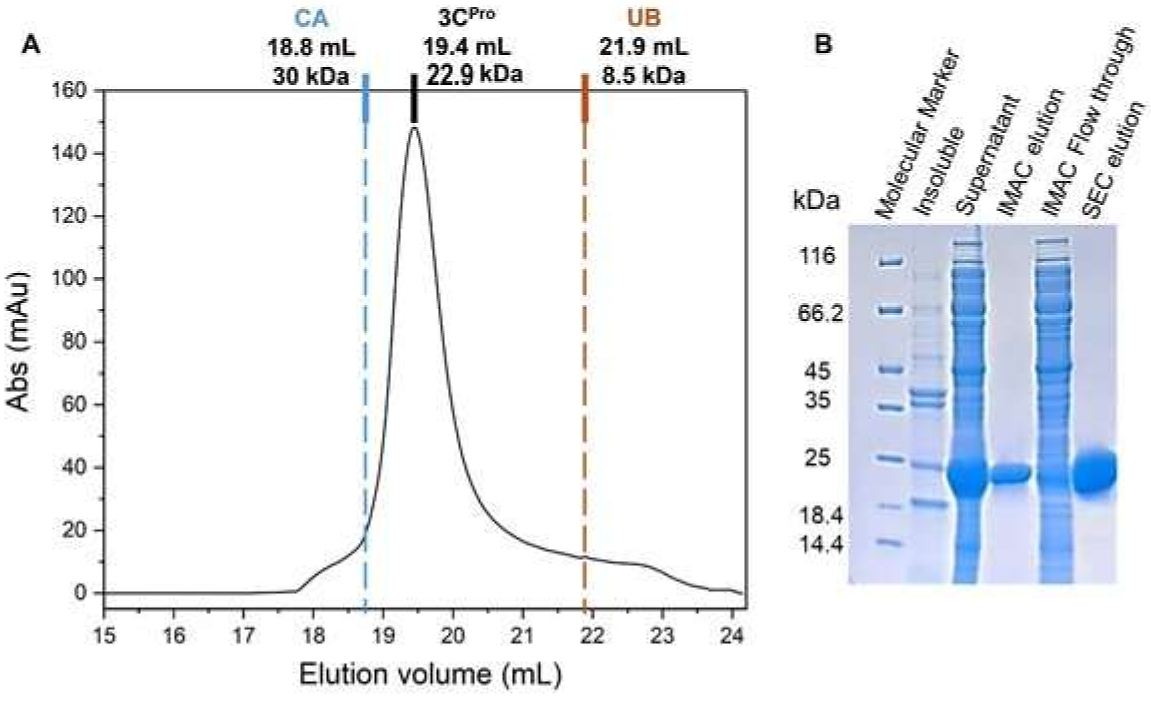

Figure S3. Solution NMR spectra acquired at 500 MHz of CVB3 $3C^{Pro}$ and of CVB3 $3C^{Pro}$ bound to GC-376 PROTAC precursor. ^{1}H-^{15}N HSQC and 3D CBCA(CO)NH spectra of CVB3 $3C^{Pro}$ (**A** and **B**), acquired at 298 K in 50 mM phosphate buffer,100 mM NaCl at pH 6.0 and 10% (v/v) D_2O, and of CVB3 $3C^{Pro}$ bound to GC-376 PROTAC precursor (**C** and **D**), acquired at 308 K in 50 mM phosphate buffer at pH 6.0 containing 100 mM NaCl, 50 mM arginine, 50 mM glutamate, 1 mM DTT and 1 mM EDTA and 10% (v/v) D_2O.

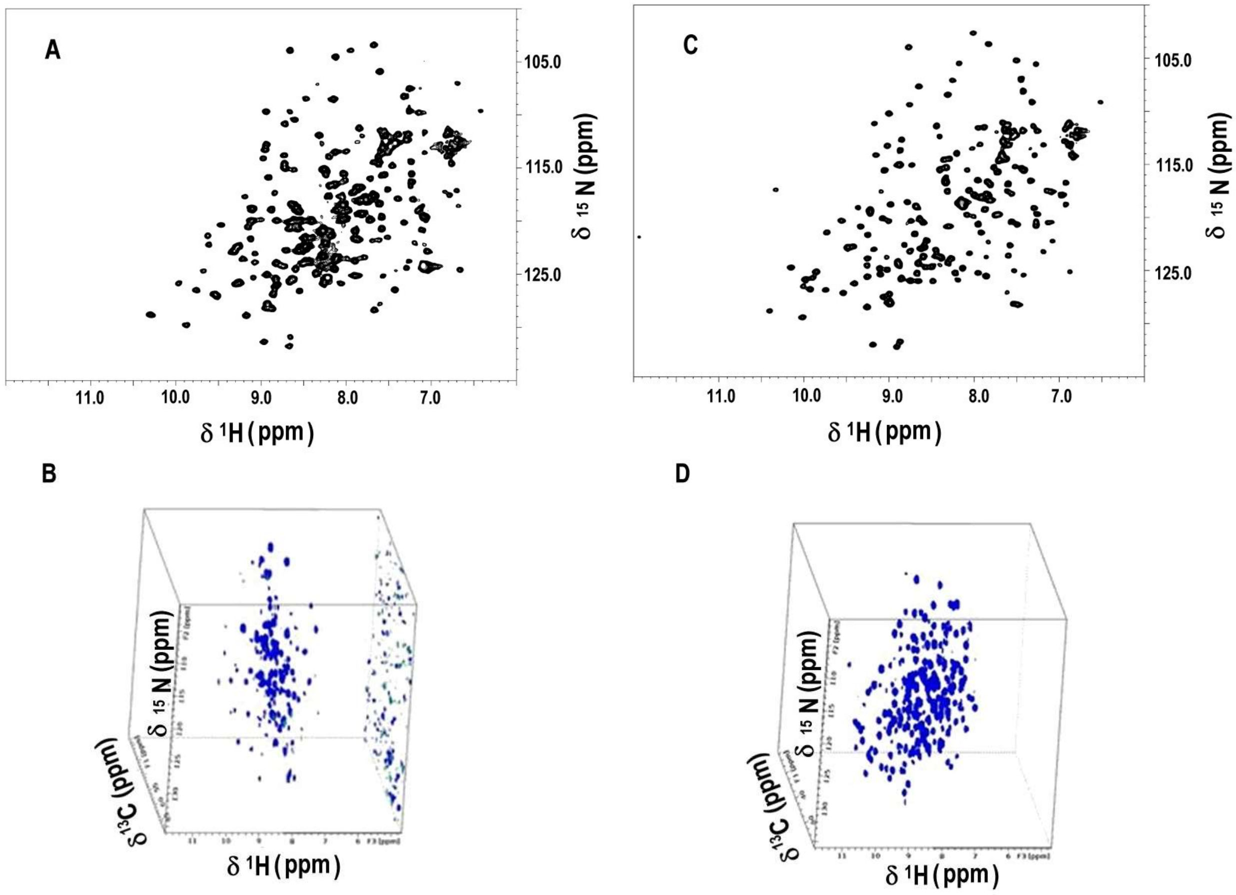

Figure S4. ^{15}N relaxation data of CVB3 3C^{Pro}. ^{15}N longitudinal (R_1) and transverse (R_2) relaxation rates and [^1H]^{15}N heteronuclear NOE values determined at 500 MHz and 298 K in a 50 mM phosphate buffer at pH 6.0 containing 100 mM NaCl, 50 mM arginine, 50 mM glutamate, 1 mM DTT and 1 mM EDTA. The secondary structure elements are shown and the position of the catalytic triad is indicated with a star.

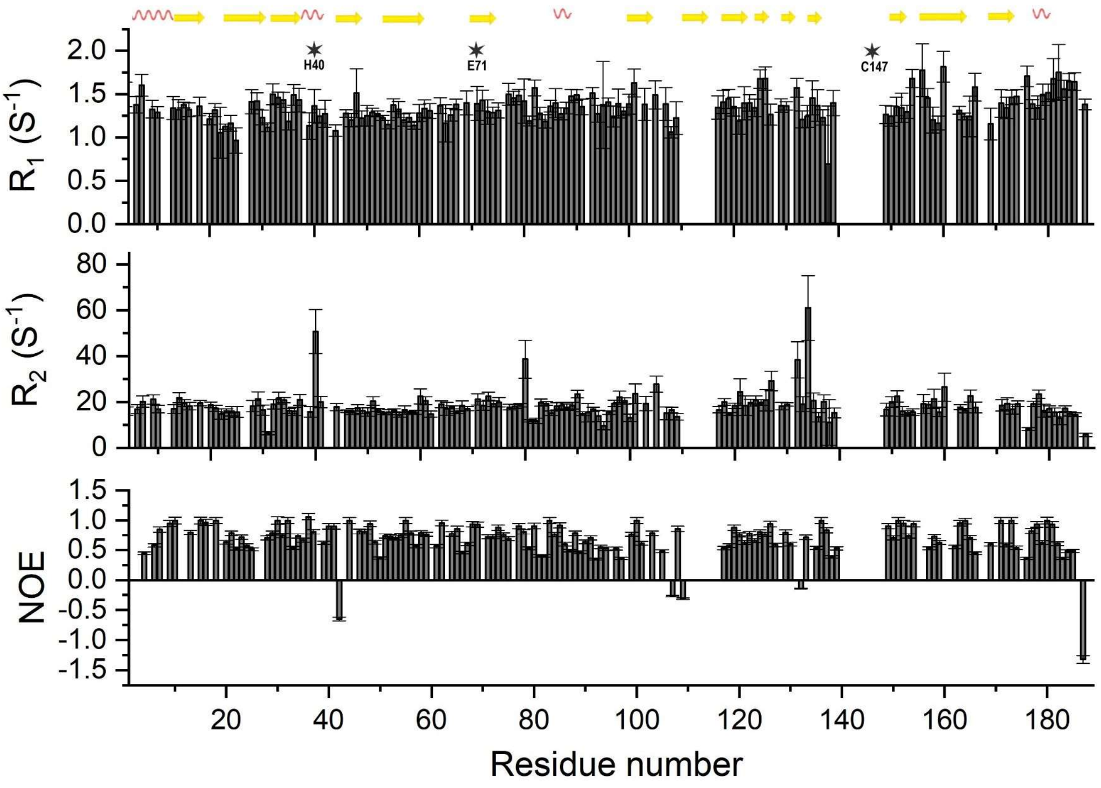

Figure S5. Monitoring the interaction of CVB3 3C^{Pro} with GC-376 PROTAC precursor and GC-376 PROTAC by solution NMR. Backbone weighted average chemical shift differences ($\Delta\delta_{avg}$(HN)) between ^{15}N-labelled CVB3 3C^{Pro} and its 1:1 mixture with GC-376 PROTAC precursor (**A**) and with GC-376 PROTAC (**B**). A chemical shift threshold value of 0.14 ppm, indicated as a dashed line in both panels, was estimated to define the most significant chemical shift differences (see the Materials and Methods section for details). The cyan (in A) and the magenta (in B) bars identify the residues having $\Delta\delta_{avg}$(HN) larger than the threshold value in the two titrations. The corresponding residues are shown as cyan and magenta spheres in Figures 5A and 5B) and are listed in Table S3. Light green and orange bars with $\Delta\delta_{avg}$(HN) = 1 indicate residues whose backbone NH signals are not assigned in the CVB3 3C^{Pro} and appear with increasing intensities along the stepwise additions of the GC-376 PROTAC precursor (light green) or GC-376 PROTAC (orange), respectively. At the end of the titrations their chemical shifts correspond to those of the GC-376 PROTAC precursor-bound or GC-376 PROTAC bound species. The backbone NHs of the latter residues are also identified as light green and orange spheres in Figure 5A and 5B, respectively. These residues are also listed in Table S4.

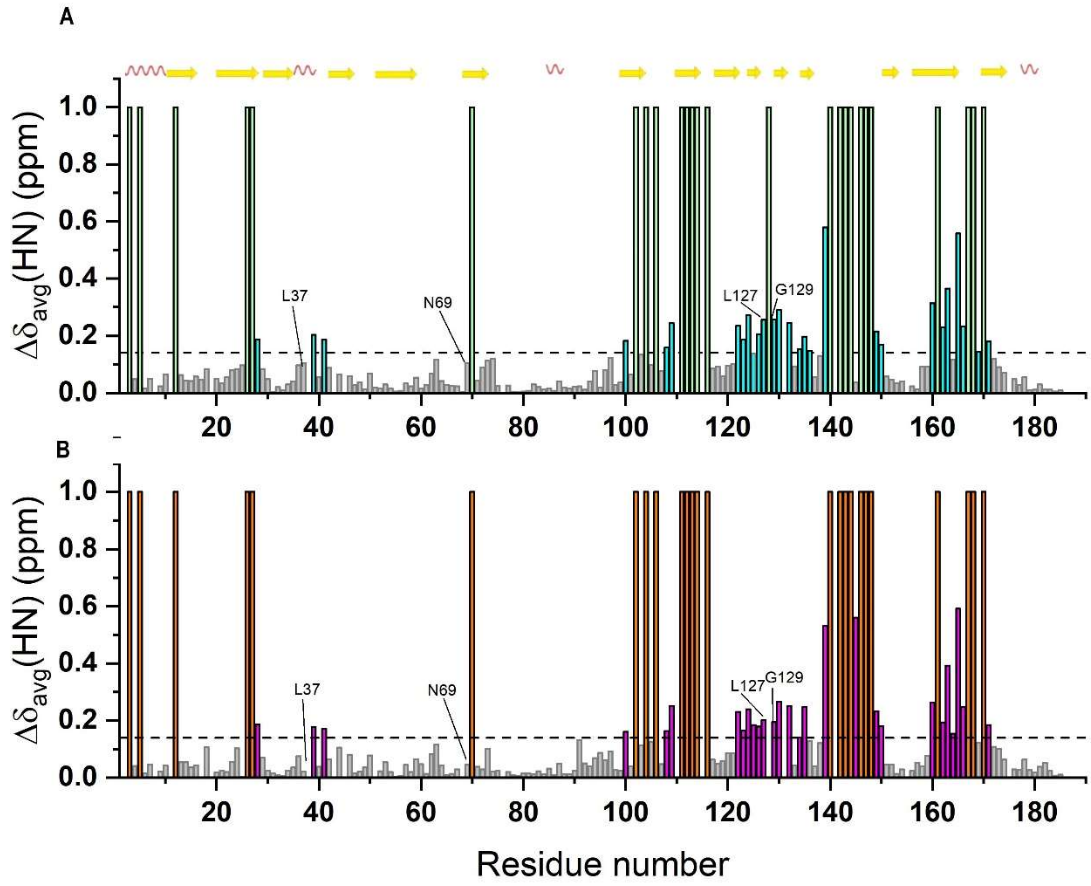

Figure S6. Comparing the interaction of CVB3 3C^{Pro} with GC-376 PROTAC and its precursor by solution NMR. Subtraction of the backbone weighted average chemical shift differences ($\Delta\Delta\delta_{avg}$(HN)) obtained between ^{15}N-labelled CVB3 3C^{Pro} and its 1:1 mixture with GC-376 PROTAC precursor and between ^{15}N-labelled CVB3 3C^{Pro} and its 1:1 mixture with GC-376 PROTAC.

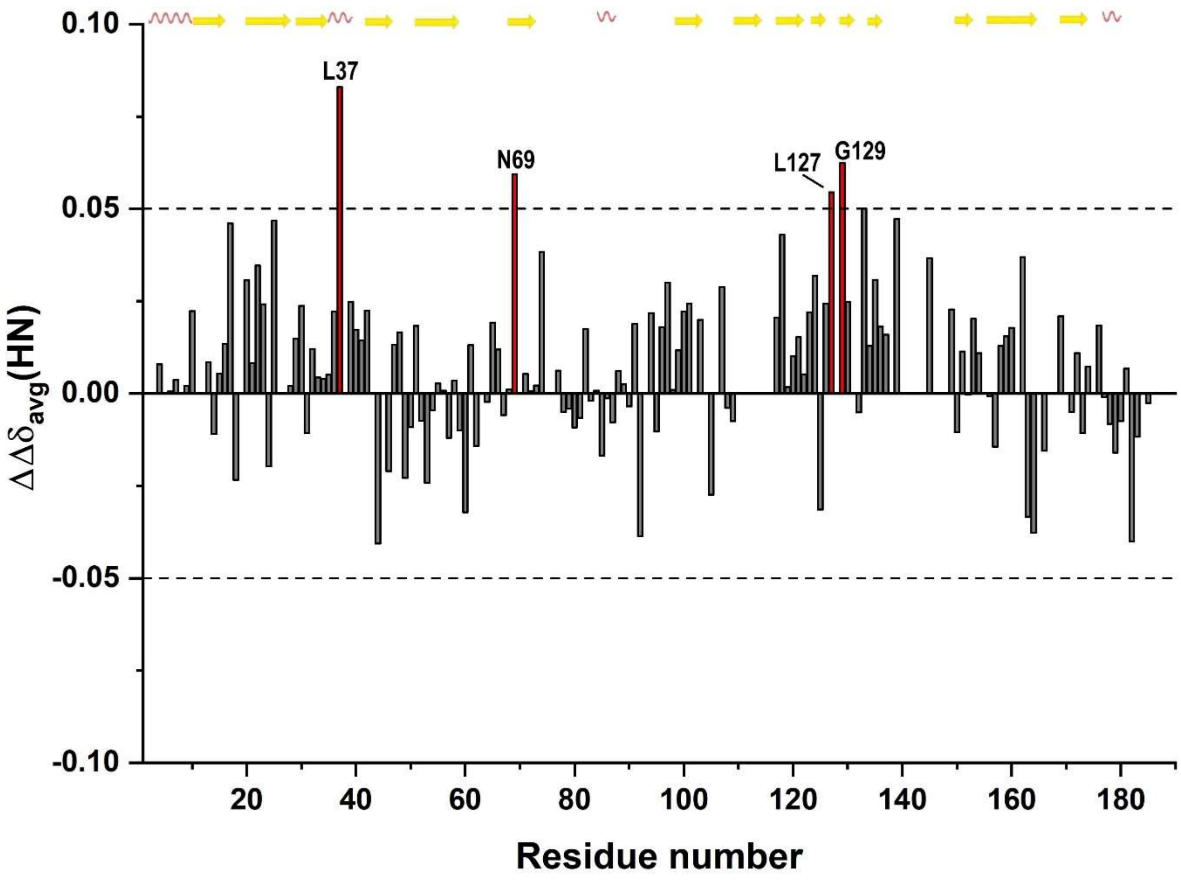

Figure S7. Representative kinetic curves of a control experiment in comparison with an inhibition experiment. Green points and line indicate the inhibition experiment, while the red ones the control experiment.

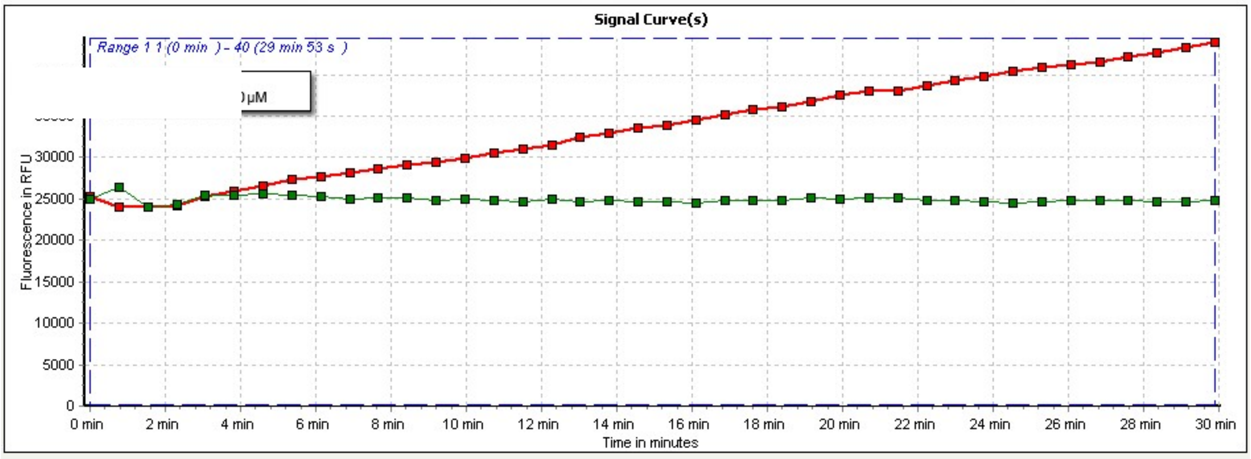

Figure S8. The raw data of enzyme inhibition kinetics of GC-376 PROTAC precursor.

Well Row	Well Col	Content	Raw Data (485-12, 520) 1 - 0 min	Raw Data (485-12, 520) 2 - 0 min 46 s	Raw Data (485-12, 520) 3 - 1 min 31 s	Raw Data (485-12, 520) 4 - 2 min 17 s	Raw Data (485-12, 520) 5 - 3 min 3 s	Raw Data (485-12, 520) 6 - 3 min 50 s	Raw Data (485-12, 520) 7 - 4 min 35 s	Raw Data (485-12, 520) 8 - 5 min 21 s	Raw Data (485-12, 520) 9 - 6 min 7 s	Raw Data (485-12, 520) 10 - 6 min 53 s
			0	0,766667	1,533333	2,3	3,066667	3,833333	4,6	5,366667	6,133333	6,9
G	1	Sample X1	6740	7212	7355	7297	7248	7429	7655	7989	8264	8572
G	2	Sample X2	6899	6486	6771	6708	6730	6869	6834	6804	6724	6714
G	3	Sample X3	5373	5797	6113	5968	5884	5828	5757	5843	5969	5927
G	4	Sample X4	5755	5778	5941	6140	6125	6058	6056	6131	6169	6233
G	5	Sample X5	5560	5555	5604	5623	5662	5755	5881	5980	6131	6224
G	6	Sample X6	6675	6102	5910	5556	5451	5533	5705	6008	6100	6374
G	7	Sample X7	6602	6331	6283	6255	6242	6325	6415	6580	6753	7002
G	8	Sample X8	6546	6467	6523	6523	6544	6730	6872	6887	7224	7439
G	9	Sample X9	5451	5334	5279	5219	5331	5451	5592	5775	5927	6021
G	10	Sample X10	6022	6405	6589	6907	7062	7224	7379	7608	7761	7888
H	1	Sample X11	5920	5382	5441	5584	5728	5869	6166	6338	6605	6745
H	2	Sample X12	6336	6232	5993	5793	5620	5715	5862	5919	5972	6077
H	3	Sample X13	6543	5949	5866	5964	5962	6030	6080	6095	6051	6132
H	4	Sample X14	5683	5257	5339	5363	5479	5505	5593	5614	5657	5761
H	5	Sample X15	5936	5557	5659	5559	5689	5624	5744	5794	5791	5832
H	6	Sample X16	6302	6029	6309	6565	6564	6367	6344	6422	6355	6357
H	7	Sample X17	5510	5531	5567	5666	5683	5754	5845	5976	6175	6298
H	8	Sample X18	5567	5531	5655	5683	5753	5780	5874	5935	6091	6263
H	9	Sample X19	4660	4749	4902	5077	5289	5413	5670	5839	5933	6192
H	10	Sample X20	5505	5701	5749	5660	5795	5874	5971	6111	6272	6372

Well Row	Well Col	Content	Raw Data (485-12, 520) 11 - 7 min 40 s	Raw Data (485-12, 520) 12 - 8 min 26 s	Raw Data (485-12, 520) 13 - 9 min 11 s	Raw Data (485-12, 520) 14 - 9 min 58 s	Raw Data (485-12, 520) 15 - 10 min 43 s	Raw Data (485-12, 520) 16 - 11 min 30 s	Raw Data (485-12, 520) 17 - 12 min 15 s	Raw Data (485-12, 520) 18 - 13 min 1 s	Raw Data (485-12, 520) 19 - 13 min 47 s	Raw Data (485-12, 520) 20 - 14 min 33 s
			7,666667	8,433333	9,2	9,966667	10,73333	11,5	12,26667	13,03333	13,8	14,56667
G	1	Sample X1	8866	9068	9601	10046	10290	10614	10744	10977	11094	11232
G	2	Sample X2	6703	6676	6653	6605	6631	6528	6590	6551	6599	6445
G	3	Sample X3	5859	5909	5882	5945	5954	5984	5924	6000	6074	6046
G	4	Sample X4	6377	6348	6354	6449	6376	6459	6540	6618	6702	6845
G	5	Sample X5	6395	6450	6556	6658	6693	6825	6900	7027	7677	7660
G	6	Sample X6	6533	6669	6801	6940	7019	7148	7252	7479	7725	7848
G	7	Sample X7	7000	7167	7322	7469	7585	7826	7944	8109	8284	8562
G	8	Sample X8	7627	7692	7969	8035	8074	8418	8561	8750	8989	9049
G	9	Sample X9	6208	6276	6482	6623	6827	7000	6964	7184	7373	7410
G	10	Sample X10	8024	8228	8391	8509	8709	8843	8986	9158	9315	9521
H	1	Sample X11	6899	7020	7034	7255	7419	7595	7771	7939	8047	8257
H	2	Sample X12	6102	6104	6106	6164	6122	6160	6151	6172	6123	6106
H	3	Sample X13	6118	6218	6187	6151	6201	6259	6273	6445	6421	6402
H	4	Sample X14	5755	5852	5879	5927	6037	6110	6294	6309	6359	6439
H	5	Sample X15	5876	5908	5958	6036	6111	6148	6251	6382	6476	6538
H	6	Sample X16	6339	6446	6455	6404	6477	6478	6477	6489	6528	6545
H	7	Sample X17	6532	6637	6830	7041	7192	7453	7615	7739	7940	8088
H	8	Sample X18	6399	6550	6744	6805	7112	7150	7224	7359	7585	7656
H	9	Sample X19	6453	6642	6839	6981	7089	7257	7353	7579	7764	7860
H	10	Sample X20	6460	6671	6868	7123	7207	7291	7371	7550	7575	7781

Well Row	Well Col	Content	Raw Data (485-12, 520) 21 - 15 min 20 s	Raw Data (485-12, 520) 22 - 16 min 6 s	Raw Data (485-12, 520) 23 - 16 min 52 s	Raw Data (485-12, 520) 24 - 17 min 37 s	Raw Data (485-12, 520) 25 - 18 min 23 s	Raw Data (485-12, 520) 26 - 19 min 10 s	Raw Data (485-12, 520) 27 - 19 min 56 s	Raw Data (485-12, 520) 28 - 20 min 41 s	Raw Data (485-12, 520) 29 - 21 min 27 s	Raw Data (485-12, 520) 30 - 22 min 14 s
			15,33333	16,1	16,86667	17,63333	18,4	19,16667	19,93333	20,7	21,46667	22,23333
G	1	Sample X1	11324	11434	11559	11738	11881	12101	12234	12354	12532	12729
G	2	Sample X2	6619	6554	6611	6577	6562	6569	6493	6537	6576	6530
G	3	Sample X3	6107	6074	6094	6202	6146	6084	6144	6203	6113	6133
G	4	Sample X4	6981	7078	7126	7160	7109	7140	7210	7147	7071	7119
G	5	Sample X5	7901	7950	7880	8053	8091	8125	8173	8191	8173	8332
G	6	Sample X6	8097	8216	8334	8360	8549	8565	8736	8734	8802	8864
G	7	Sample X7	8771	9034	9167	9289	9501	9660	9547	9756	9806	9939
G	8	Sample X8	9260	9396	9593	9725	9908	10033	10102	10237	10355	10398
G	9	Sample X9	7579	7630	7783	7852	7938	8091	8170	8274	8315	8453
G	10	Sample X10	9636	9789	9893	9947	10180	10273	10466	10472	10706	10771
H	1	Sample X11	8359	8510	8863	8949	9133	9174	9438	9508	9675	9730
H	2	Sample X12	6060	6118	6183	6108	6132	6110	6077	6052	6000	6033
H	3	Sample X13	6469	6478	6496	6474	6492	6484	6549	6507	6474	6541
H	4	Sample X14	6528	6523	6535	6567	6548	6578	6541	6551	6602	6608
H	5	Sample X15	6666	6736	6833	6842	6889	6860	6930	6876	6890	6959
H	6	Sample X16	6571	6585	6607	6533	6668	6724	6631	6606	6626	6664
H	7	Sample X17	8248	8380	8534	8678	8875	9026	9021	9261	9281	9515
H	8	Sample X18	7723	7979	8178	8431	8471	8598	8769	8940	9080	9216
H	9	Sample X19	7881	8000	8029	8171	8378	8374	8482	8798	8843	9047
H	10	Sample X20	7985	8197	8274	8498	8688	8765	8994	9032	9260	9397

Well Row	Well Col	Content	Raw Data (485-12, 520) 31 - 23 min	Raw Data (485-12, 520) 32 - 23 min 45 s	Raw Data (485-12, 520) 33 - 24 min 31 s	Raw Data (485-12, 520) 34 - 25 min 18 s	Raw Data (485-12, 520) 35 - 26 min 3 s	Raw Data (485-12, 520) 36 - 26 min 49 s	Raw Data (485-12, 520) 37 - 27 min 35 s	Raw Data (485-12, 520) 38 - 28 min 22 s	Raw Data (485-12, 520) 39 - 29 min 7 s	Raw Data (485-12, 520) 40 - 29 min 53 s
			23	23,76667	24,53333	25,3	26,06667	26,83333	27,6	28,36667	29,13333	29,9
G	1	Sample X1	12799	12797	13124	13345	13239	13482	13580	13773	13895	14042
G	2	Sample X2	6552	6474	6568	6587	6510	6517	6546	6493	6522	6539
G	3	Sample X3	6149	6191	6176	6247	6210	6235	6174	6131	6223	6189
G	4	Sample X4	7082	7175	7158	7197	7190	7202	7237	7240	7164	7205
G	5	Sample X5	8399	8384	8494	8472	8524	8483	8665	8666	8597	8681
G	6	Sample X6	8998	8972	9051	9230	9333	9343	9516	9479	9628	9724
G	7	Sample X7	10159	10192	10334	10442	10560	10704	10719	10930	10906	11214
G	8	Sample X8	10542	10744	10926	10956	11029	11302	11306	11552	11579	11830
G	9	Sample X9	8536	8713	8804	8922	9025	9154	9256	9365	9517	9531
G	10	Sample X10	10808	10952	11056	11240	11346	11516	11707	11762	11870	12024
H	1	Sample X11	9919	10024	10117	10269	10346	10600	10511	10837	11044	11081
H	2	Sample X12	6046	6110	6069	6058	6078	6029	6082	6029	6087	6047
H	3	Sample X13	6464	6553	6508	6559	6580	6559	6557	6581	6502	6561
H	4	Sample X14	6647	6568	6633	6647	6647	6616	6666	6616	6681	6685
H	5	Sample X15	6971	6921	7000	7106	6964	7059	7128	7236	7098	7112
H	6	Sample X16	6663	6585	6597	6649	6634	6613	6642	6594	6631	6623
H	7	Sample X17	9721	9721	10021	10149	10268	10391	10420	10569	10700	10810
H	8	Sample X18	9430	9508	9623	9837	9964	10108	10158	10254	10382	10495
H	9	Sample X19	9104	9135	9328	9525	9623	9707	9919	10014	10130	10306
H	10	Sample X20	9546	9682	10006	9959	10093	10246	10351	10538	10672	10758

Figure S9. Raw data of calculated inhibition curve of GC-376 PROTAC precursor.

Well Row	Well Col	Content	Raw Data (485-12, 520) 1 - 0 min	Linear regression fit of Range 1 based on Raw Data (485-12, 520) 1 - 0 min	Slope of Linear regression fit (Range 1) based on Raw Data (485-12, 520)	r^2 of Linear regression fit (Range 1) based on Raw Data (485-12, 520)	% Inhibition (calculated using the formula 100-(v1/vo)* 100	Inhibitor concentration				Log[M]	% Inhibition	% Inhibition
			0	0										
G	1	Sample X1	6740	6740	4,142022	0,969915	ctrl	0	3,733627	average ctrl		-4	103,732	97,9772
G	2	Sample X2	6899	6899	-0,13934	0,502185	103,732	100 µM				-4,5	93,90662	89,4247
G	3	Sample X3	5373	5373	0,227504	0,743477	93,90662	30 µM				-5	77,28203	77,5484
G	4	Sample X4	5755	5755	0,848205	0,893319	77,28203	10 µM				-5,5	45,77699	42,69942
G	5	Sample X5	5560	5560	2,024485	0,946434	45,77699	3 µM				-6	31,51275	40,99121
G	6	Sample X6	6675	6675	2,557059	0,969077	31,51275	1 µM				-6,5	16,97036	12,48713
G	7	Sample X7	6602	6602	3,100018	0,988537	16,97036	300 nM				-7	13,5861	18,27182
G	8	Sample X8	6546	6546	3,226373	0,994169	13,5861	100 nM				-8	17,36143	17,98093
G	9	Sample X9	5451	5451	3,085416	0,994116	17,36143	10 nM				-9	16,51646	16,20282
G	10	Sample X10	6022	6022	3,116964	0,993718	16,51646	1 nM						
H	1	Sample X11	5920	5920	3,325233	0,996018	ctrl	0						
H	2	Sample X12	6336	6336	0,075524	0,102954	97,9772	100 µM						
H	3	Sample X13	6543	6543	0,394842	0,875302	89,4247	30 µM						
H	4	Sample X14	5683	5683	0,838259	0,874699	77,5484	10 µM						
H	5	Sample X15	5936	5936	2,13939	0,943858	42,69942	3 µM						
H	6	Sample X16	6302	6302	0,203168	0,606018	94,55842	1 µM						
H	7	Sample X17	5510	5510	3,267405	0,995398	12,48713	300 nM						
H	8	Sample X18	5567	5567	3,051426	0,99541	18,27182	100 nM						
H	9	Sample X19	4660	4660	3,062287	0,98938	17,98093	10 nM						
H	10	Sample X20	5505	5505	3,128675	0,995616	16,20282	1 nM						

Figure S10. Raw data of enzyme inhibition kinetics of GC-376 PROTAC.

Well Row	Well Col	Content	Raw Data (485-12, 520) 1 - 0 min	Raw Data (485-12, 520) 2 - 0 min 45 s	Raw Data (485-12, 520) 3 - 1 min 30 s	Raw Data (485-12, 520) 4 - 2 min 15 s	Raw Data (485-12, 520) 5 - 3 min	Raw Data (485-12, 520) 6 - 3 min 45 s	Raw Data (485-12, 520) 7 - 4 min 30 s	Raw Data (485-12, 520) 8 - 5 min 15 s	Raw Data (485-12, 520) 9 - 6 min	Raw Data (485-12, 520) 10 - 6 min 45 s
			0	0,75	1,5	2,25	3	3,75	4,5	5,25	6	6,75
A	1	Sample X1	4383	4719	4881	5069	5233	5418	5720	6018	6404	6738
A	2	Sample X2	6743	6729	6958	7246	7688	8087	8430	8614	8906	9145
A	3	Sample X3	4911	5017	5079	5242	5431	5721	6038	6358	6538	6868
A	4	Sample X4	5200	5043	5003	5078	5203	5482	5814	6018	6344	6665
A	5	Sample X5	4532	4540	4664	4886	5176	5522	5870	6200	6514	6829
A	6	Sample X6	4411	4366	4467	4660	5002	5354	5701	6096	6317	6690
A	7	Sample X7	4187	4086	4316	4625	4946	5283	5657	6003	6205	6503
A	8	Sample X8	4605	4803	5126	5418	5748	6139	6486	6843	7186	7600
A	9	Sample X9	4533	4816	5073	5325	5690	5928	6296	6640	6936	7295
A	10	Sample X10	4371	4676	5031	5380	5771	6214	6529	6851	7145	7489
B	1	Sample X12	4137	3896	3936	4129	4308	4632	4870	5029	5078	5238
B	2	Sample X13	7175	7214	7092	7267	7491	7731	7873	7990	8222	8338
B	3	Sample X14	5019	4885	4999	5178	5432	5665	6068	6285	6616	6875
B	4	Sample X15	4810	4497	4725	4949	5234	5555	5947	6246	6519	6785
B	5	Sample X16	4351	4268	4602	5011	5265	5556	5907	6171	6381	6607
B	6	Sample X17	4550	4436	4765	5071	5429	5809	6204	6548	6804	7162
B	7	Sample X18	4431	4610	4957	5339	5684	5979	6319	6687	6950	7405
B	8	Sample X19	4405	4643	4925	5286	5610	5849	6269	6572	7082	7515
B	9	Sample X20	4122	4328	4732	5104	5482	5787	6160	6555	6831	7108
B	10	Sample X21	4470	4490	4910	5372	5716	6048	6417	6730	7061	7465
C	1	Sample X23	3822	3679	3860	4022	4147	4453	4600	4824	5031	5101
C	2	Sample X24	7362	7591	7516	7603	7820	8121	8429	8733	8926	9239
C	3	Sample X25	5261	5180	5342	5505	5831	6052	6316	6661	6867	7096
C	4	Sample X26	5229	4948	4878	4875	5077	5318	5655	5855	6044	6270
C	5	Sample X27	5137	4804	4990	5232	5537	5832	6302	6568	6887	7206
C	6	Sample X28	4172	4226	4536	5027	5340	5580	5963	6239	6582	6740
C	7	Sample X29	4398	4703	5003	5287	5585	5954	6446	6681	6977	7314
C	8	Sample X30	4779	5020	5299	5602	5767	6058	6410	6583	6967	7248
C	9	Sample X31	4414	4633	4987	5435	5701	6119	6403	6743	7043	7437
C	10	Sample X32	3662	3953	4315	4714	5015	5239	5465	5714	5942	6139

Well Row	Well Col	Content	Raw Data (485-12, 520) 11 - 7 min 30 s	Raw Data (485-12, 520) 12 - 8 min 15 s	Raw Data (485-12, 520) 13 - 9 min	Raw Data (485-12, 520) 14 - 9 min 45 s	Raw Data (485-12, 520) 15 - 10 min 30 s	Raw Data (485-12, 520) 16 - 11 min 15 s	Raw Data (485-12, 520) 17 - 12 min	Raw Data (485-12, 520) 18 - 12 min 45 s	Raw Data (485-12, 520) 19 - 13 min 30 s	Raw Data (485-12, 520) 20 - 14 min 15 s
			7,5	8,25	9	9,75	10,5	11,25	12	12,75	13,5	14,25
A	1	Sample X1	7101	7297	7635	7781	8100	8308	8592	8788	8982	9312
A	2	Sample X2	9359	9449	9656	9845	10134	10236	10477	10729	10746	11014
A	3	Sample X3	7171	7367	7613	7919	8048	8432	8619	8724	8983	9262
A	4	Sample X4	6955	7238	7458	7833	7899	8188	8461	8731	9240	9520
A	5	Sample X5	7210	7456	7735	8039	8270	8591	8808	9120	9294	9666
A	6	Sample X6	7028	7311	7575	7770	8077	8331	8501	8759	9019	9170
A	7	Sample X7	6728	6929	7209	7402	7707	7963	8182	8321	8660	8754
A	8	Sample X8	7834	8099	8487	8827	9137	9433	9782	10077	10441	10662
A	9	Sample X9	7707	7965	8122	8505	8832	9162	9414	9644	9941	10138
A	10	Sample X10	7864	8205	8546	8932	9160	9483	9783	10190	10330	10584
B	1	Sample X12	5381	5552	5650	5824	5896	6013	6205	6209	6356	6449
B	2	Sample X13	8582	8519	8723	8837	8996	9083	9225	9398	9466	9676
B	3	Sample X14	7138	7316	7602	7839	8055	8222	8458	8695	8948	9137
B	4	Sample X15	7066	7260	7624	7865	8184	8540	8760	9019	9363	9612
B	5	Sample X16	6956	7129	7389	7646	7890	8180	8374	9058	9405	9578
B	6	Sample X17	7444	7656	7962	8292	8612	8958	9291	9589	9746	10157
B	7	Sample X18	7706	8038	8266	8598	8867	9235	9442	9804	10101	10271
B	8	Sample X19	7958	8109	8433	8819	9100	9359	9714	9931	10254	10481
B	9	Sample X20	7589	7800	8143	8527	8747	9179	9409	9697	9919	10123
B	10	Sample X21	7772	8014	8345	8612	8965	9279	9620	9895	10124	10328
C	1	Sample X23	5300	5445	5481	5600	5786	5895	6088	6196	6332	6439
C	2	Sample X24	9531	9678	9882	10105	10267	10527	10662	10969	11134	11398
C	3	Sample X25	7426	7659	7741	8055	8368	8605	8869	9071	9269	9615
C	4	Sample X26	6432	6655	6929	7119	7306	7548	7801	8452	8580	8797
C	5	Sample X27	7497	7768	8002	8412	8513	8955	9235	9590	9864	10121
C	6	Sample X28	7050	7415	7667	7936	8751	8993	9255	9438	9771	9998
C	7	Sample X29	7708	7958	8351	8600	8848	9056	9497	9751	10045	10300
C	8	Sample X30	7418	7767	7993	8294	8595	8922	9184	9534	9725	9949
C	9	Sample X31	7739	8047	8372	8672	8984	9251	9665	9969	10218	10608
C	10	Sample X32	6516	6711	6924	7166	7354	7568	7811	8002	8278	8634

Well Row	Well Col	Content	Raw Data (485-12, 520) 21 - 15 min	Raw Data (485-12, 520) 22 - 15 min 45 s	Raw Data (485-12, 520) 23 - 16 min 30 s	Raw Data (485-12, 520) 24 - 17 min 15 s	Raw Data (485-12, 520) 25 - 18 min	Raw Data (485-12, 520) 26 - 18 min 45 s	Raw Data (485-12, 520) 27 - 19 min 30 s	Raw Data (485-12, 520) 28 - 20 min 15 s	Raw Data (485-12, 520) 29 - 21 min	Raw Data (485-12, 520) 30 - 21 min 45 s
			15	15,75	16,5	17,25	18	18,75	19,5	20,25	21	21,75
A	1	Sample X1	9514	9848	10166	10366	10663	10916	11196	11529	11725	12032
A	2	Sample X2	11342	11516	11632	11935	12064	12238	12484	12745	12877	13184
A	3	Sample X3	9531	9758	10107	10271	10581	10825	11073	11307	11422	11729
A	4	Sample X4	9835	10034	10238	10579	10874	11026	11412	11553	11753	12033
A	5	Sample X5	10064	10389	10609	10871	11125	11535	11724	12045	12273	12472
A	6	Sample X6	9472	9595	9984	10161	10489	10696	10921	11161	11399	11641
A	7	Sample X7	9111	9224	9563	9718	9987	10173	10491	10659	10911	11085
A	8	Sample X8	10989	11247	11644	11853	12245	12417	12785	13171	13376	13779
A	9	Sample X9	10489	10775	11082	11226	11753	11935	12239	12621	12837	13054
A	10	Sample X10	10957	11251	11546	11848	12190	12569	12801	13118	13445	13579
B	1	Sample X12	6546	6594	6702	6935	6926	7051	7156	7375	7370	7533
B	2	Sample X13	9897	10001	10155	10298	10501	10725	10863	10961	11086	11362
B	3	Sample X14	9304	9537	9828	10052	10220	10568	10780	11032	11195	11498
B	4	Sample X15	9859	10067	10402	10777	11303	11625	11763	12071	12355	12558
B	5	Sample X16	9734	10013	10253	10522	10754	11135	11410	11676	11988	12125
B	6	Sample X17	10323	10689	11007	11311	11572	11828	12263	12579	12755	13008
B	7	Sample X18	10528	10938	11166	11567	11914	12214	12762	12751	13110	13357
B	8	Sample X19	10772	11076	11440	11742	12052	12407	12730	13070	13396	13619
B	9	Sample X20	10505	10819	11156	11397	11677	12017	12367	12569	12738	13065
B	10	Sample X21	10768	10952	11234	11594	11882	12120	12348	12668	12974	13205
C	1	Sample X23	6495	6559	6763	6789	7022	7163	7320	7305	7404	7576
C	2	Sample X24	11641	11839	12094	12227	12522	12822	13043	13161	13517	13692
C	3	Sample X25	9785	9944	10192	10401	10622	10963	11201	11389	11502	11859
C	4	Sample X26	9022	9200	9403	9655	9914	10089	10363	10669	10823	11055
C	5	Sample X27	10324	10606	11072	11333	11486	11814	12212	12353	12778	13102
C	6	Sample X28	10172	10552	10835	11059	11337	11728	11932	12162	12492	12809
C	7	Sample X29	10484	10919	11214	11553	11786	12187	12398	12764	13080	13321
C	8	Sample X30	10360	10648	10895	11255	11552	11928	12165	12459	12727	13081
C	9	Sample X31	10840	11350	11618	11855	12106	12523	12887	13072	13359	13701
C	10	Sample X32	8798	9003	9354	9560	9736	10008	10176	10417	10735	10963

Figure S11. Raw data of calculated inhibition curve of GC-376 PROTAC.

Well Row	Well Col	Content	Raw Data (485-12, 520) 1 - 0 min	Linear regression fit of Range 1 based on Raw Data (485-12, 520) 1 - 0 min	Slope of Linear regression fit (Range 1) based on Raw Data (485-12, 520)	r^2 of Linear regression fit (Range 1) based on Raw Data (485-12, 520)	% Inhibition (calculated using the formula 100-(v1/vo)*100	Inhibitor concentration				Log[M]	% Inhibition	% Inhibition	% Inhibition
			0	0											
A	1	Sample X1	4383	4383	8,189947	0,97291	ctrl	0	8,08531	average ctrl		-3,5	47,22549	55,49012	40,42337
A	2	Sample X2	6743	6743	4,267196	0,991048	47,22286	300 µM				-4	35,47965	29,8194	31,10195
A	3	Sample X3	4911	4911	5,216931	0,972472	35,47643	100 µM				-4,5	45,46198	*17,96231**	50,47114
A	4	Sample X4	5200	5200	4,409788	0,917966	45,46198	30 µM				-5	18,87188	16,19225	15,01112
A	5	Sample X5	4532	4532	6,559788	0,986794	18,87188	10 µM				-6	17,29813	4,233739	8,209004
A	6	Sample X6	4411	4411	6,687037	0,982113	17,29813	1 µM				-7	12,63905	7,521921	7,885094
A	7	Sample X7	4187	4187	7,063757	0,996297	12,63905	100 nM				-8	5,571915	6,76613	*25,52022**
A	8	Sample X8	4605	4605	7,635185	0,998848	5,571915	10 nM				-9	15,5019	1,48868	5,208742
A	9	Sample X9	4533	4533	6,832275	0,997156	15,5019	1 nM				-10	0,952101	0	*23,4557**
A	10	Sample X10	4371	4371	8,00873	0,997394	0,952101	0.1 nM							
B	1	Sample X12	4137	4137	8,308466	0,972267		0				**data excluded for the fitting*			
B	2	Sample X13	7175	7175	3,598942	0,949898	55,49012	300 µM							
B	3	Sample X14	5019	5019	5,674603	0,981609	29,8194	100 µM							
B	4	Sample X15	4810	4810	6,633333	0,993902	17,96231	30 µM							
B	5	Sample X16	4351	4351	6,776455	0,994843	16,19225	10 µM							
B	6	Sample X17	4550	4550	7,743386	0,998126	4,233739	1 µM							
B	7	Sample X18	4431	4431	7,477513	0,998734	7,521921	100 nM							
B	8	Sample X19	4405	4405	7,538624	0,993911	6,76613	10 nM							
B	9	Sample X20	4122	4122	7,965344	0,998676	1,488679	1 nM							
B	10	Sample X21	4470	4470	8,085714	0,996345	0	0.1 nM							
C	1	Sample X23	3822	3822	7,757519	0,993549	4,058958	0							
C	2	Sample X24	7362	7362	4,817196	0,932391	40,42337	300 µM							
C	3	Sample X25	5261	5261	5,570899	0,991348	31,10195	100 µM							
C	4	Sample X26	5229	5229	4,004762	0,901882	50,47114	30 µM							
C	5	Sample X27	5137	5137	6,871958	0,990076	15,01112	10 µM							
C	6	Sample X28	4172	4172	7,421958	0,995291	8,209004	1 µM							
C	7	Sample X29	4398	4398	7,448148	0,994639	7,885094	100 nM							
C	8	Sample X30	4779	4779	6,022222	0,994965	25,52022	10 nM							
C	9	Sample X31	4414	4414	7,66455	0,997176	5,208742	1 nM							
C	10	Sample X32	3662	3662	6,189153	0,986633	23,4557	0.1 nM							
